# Acceptor‐Doped Cerium Oxides Enabling Metallic Nanocatalyst Exsolution for Hydrogen Production From Ammonia

**DOI:** 10.1002/anie.6182981

**Published:** 2026-07-24

**Authors:** Andrés López‐García, Elena Barrio‐Querol, Álvaro Represa, Jesús Ara, Ana B. Hungría, David Catalán‐Martínez, Alfonso J. Carrillo, Sonia Escolástico, José Manuel Serra

**Affiliations:** ^1^ Instituto de Tecnología Química (Consejo Superior De Investigaciones Científicas ‐ Universitat Politècnica de València) València Spain; ^2^ Departamento de Ciencia de Materiales Ingeniería Metalúrgica y Química Inorgánica Campus Río San Pedro Universidad De Cádiz Puerto Real Spain

**Keywords:** ammonia decomposition, catalyst, ceria, exsolution, hydrogen production, metal nanoparticles, Ru

## Abstract

The development of advanced catalysts with enhanced activity and durability is essential for driving sustainable hydrogen production through ammonia cracking. In this sense, exsolution has gained prominence for generating stable and active nanoparticles socketed into the oxide support with high resistance to sintering. However, challenges remain in expanding these methods to CeO_2_‐based systems due to the limited solubility of transition metals in fluorite oxides. Here, it is demonstrated that incorporating Gd into the CeO_2_ lattice enhances the successful dissolution of Ru and Rh, promoting the exsolution of highly dispersed metallic nanoparticles (∼3 nm), including RuRh alloys. The resulting Ru‐exsolved@Ce_0.8_Gd_0.2_O_2‐δ_ catalyst exhibits remarkable catalytic activity and long‐term stability in ammonia decomposition for 260 h, outperforming Ru‐impregnated catalysts. In addition, benchmarking against literature values demonstrates that the Ru‐exsolved@Ce_0.8_Gd_0.2_O_2‐δ_ are among the most active catalysts at 400 °C. This work highlights the potential of lattice modification of CeO_2_‐based catalytic supports via acceptor doping, resulting in significant metal‐exsolution dispersion and remarkably active catalysts for hydrogen generation via ammonia decomposition, with potential applications that can be expanded to other (electro)catalytic systems.

## Graphical abstract

Outstanding catalytic results for NH_3_ decomposition for H_2_ production were obtained with metallic Ru, Rh, and RuRh nanoparticle‐exsolved CeO_2_‐based catalysts. Significant lower amounts of available superficial metallic fractions were needed in comparison to conventional Ru‐impregnated materials.

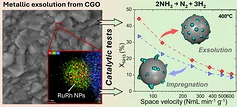

## 1 Introduction

The design and development of stable, active metal‐supported catalysts are central to advancing numerous industrial catalytic processes, especially those involving hydrogen production from carbon‐free carriers like ammonia (NH_3_) [1]. Ammonia decomposition [2] (NH_3_ cracking) has attracted significant attention as a sustainable route to generate clean hydrogen (H_2_), particularly due to ammonia's high hydrogen content (17.8 wt.%) [1, 3] and its established global production infrastructure [1, 3, 4, 5, 6, 7]. Moreover, the ease of ammonia liquefaction [8, 9] at moderate pressures offers an attractive alternative to the complex handling of hydrogen gas [10]. However, the successful commercialization of NH_3_ cracking focuses on improving the performance and durability of catalysts, which is currently limited by issues such as catalyst deactivation [11] due to nanoparticle (NP) degradation or poisoning, besides sintering under high‐temperature conditions [12].

Therefore, unlike the well‐established NH_3_ synthesis via the Haber‐Bosch process [9, 13], NH_3_ decomposition technologies are still susceptible to significant development, requiring advancements for industrial scalability in the catalyst and heat management. NH_3_ decomposition is a metal‐catalyzed, endothermic reaction [8]. Ru, Ni, and Rh are widely recognized as the most active metals for this reaction [10, 14, 15], followed by other metals, such as Co > Ir > Fe > Pt [2, 16, 17], with Ru demonstrating the highest performance. However, the high cost of Ru and its susceptibility to deactivation via high‐temperature agglomeration or volatilization present significant challenges for conventional Ru‐supported catalysts [15]. In this sense, while traditional supports like Al_2_O_3_, SiO_2_, and MgO have been employed [18, 19], CeO_2_‐based supports have gained attention due to their Strong Metal‐Support Interactions (SMSI), which mitigate sintering and enhance catalytic stability [20]. Lucentini et al. proved the superior performance of CeO_2_‐supported catalysts compared to Al_2_O_3_‐based ones [10]. Studies on ceria‐supported Ni, Ru, and Ni‐Ru bimetallic catalysts have shown promising results [2, 10, 21, 22]. However, the high operational temperatures can pose morphological limitations. In this regard, exsolution‐based catalysts offer a transformative approach for improving NH_3_ decomposition, addressing these key challenges, and unlocking NH_3_ potential as a sustainable hydrogen source. Their robust, socketed NPs overcome common limitations of conventional metal‐supported systems [23], making them a promising yet unexplored [24, 25] direction for catalyst development.

Exsolution allows the in situ generation of highly stable, anchored metal NPs on oxide supports [26, 27, 28, 29]. Unlike traditional impregnation methods, exsolved NPs remain embedded in the host oxide surface, which enhances their resistance to sintering and coke formation, ultimately improving long‐term catalytic stability [30]. Exsolution has been extensively studied in perovskite‐based oxides [31, 32, 33, 34, 35] and double perovskites [35, 36, 37, 38, 39, 40, 41], but also spinels [42, 43]. However, its application to fluorite (e.g., ZrO_2_ or CeO_2_) systems remains underdeveloped due to the limited solubility of transition metals in the lattice, which leads to the undesired formation of secondary phases [30] (e.g., metal oxides) that may hinder the exsolution process [44]. Overcoming these limitations through advanced material design to modify fluorite‐type lattice properties and improve its capacity to accommodate exsolvable cations (e.g., Ni, Co, Cu, Ru, Rh) may significantly expand their applicability to various chemical processes, particularly to high‐temperature endothermic catalytic processes. Among fluorite‐structured materials, and in line with the growing interest in the NH_3_ cracking process, CeO_2_ stands out because of its physicochemical properties, including high thermal stability and redox flexibility, making it a valuable material for thermal and electrocatalytic processes [20, 45, 46], but reports on metallic NP exsolution from CeO_2_ remain scarce.

One of the first studies in the fluorite exsolution field was conducted by Naeem et al., who obtained Ru exsolved NPs from Sm_2_Ru_x_Ce_2−x_O_7_ pyrochlores [47]. Their findings revealed that these exsolved nanocatalysts exhibited greater stability than conventional impregnation methods in dry methane reforming. Focusing on CeO_2_ material, Carrillo et al. showed the exsolution of Ru NPs [48], which improved the catalytic activity for syngas production under chemical looping methane reforming. However, Ru had to be targeted at low concentrations due to the limited solubility [48]. Other metals, including Fe [49], Ni [50], and Pt [50, 51], as well as metallic alloys such as Cu‐Ni [52] and Pt‐Ru [53], have also been exsolved from cerium oxides.

**FIGURE 1:**
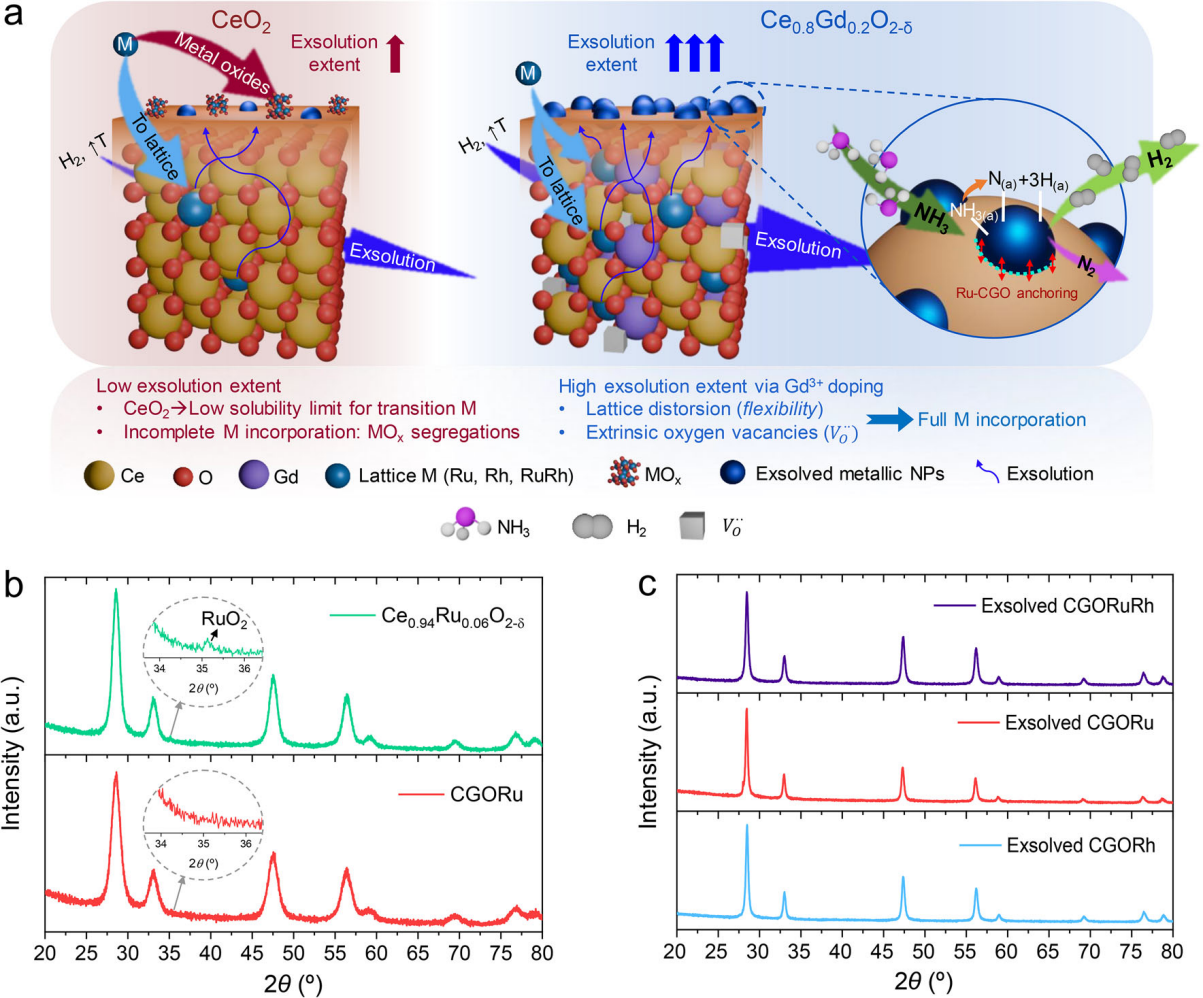
Schematic representation of the implications of introducing Gd^3+^ in the CeO_2_ lattice. Partial substitution of Ce cations with acceptor dopant Gd^3+^ facilitates the allocation of transition metals (M) in the lattice. This full incorporation of M (Ru, Rh, or both in this work) results in higher metallic NP exsolution extents after reduction treatment. In contrast, adding equal amounts of transition M in undoped CeO_2_ results in incomplete incorporation and subsequent segregation of metal oxides (MO_x_), hampering the extent of exsolution. The CGO‐doped exsolved catalyst offered strong anchoring between CGO and metallic NPs for the NH_3_ decomposition reaction, imparting remarkable catalytic activity and stability (a). PXRD diffractograms of the pristine CGORu material (700 °C, 5 h in air) compared to Ce_0.94_Ru_0.06_O_2__‐δ_ prepared under the same conditions (b). RuO_2_ segregations can be appreciated in the absence of Gd^3+^, conversely to CGORu material. PXRD diffractograms of CGO materials (CGORuRh, CGORu, and CGORh) after reduction treatments for exsolution (700 °C, 4 h under 5% H_2_/Ar flow) (c).

In this work, the exsolution of Ru and Rh NPs from Gd‐doped cerium oxide (CGO) supports is explored. By partially substituting Ce with an acceptor dopant like Gd, the incorporation of Ru and Rh into the lattice is facilitated, resulting in an increase compared to CeO_2_, and avoiding metal oxide segregations, thereby facilitating their subsequent exsolution (Figure 1a). The exsolved Ru, Rh, and RuRh (to explore the potential synergistic effect of RuRh‐alloyed NPs) catalysts were tested for NH_3_ decomposition under different temperature and space velocity conditions, allowing kinetic modeling studies on them. These exsolved catalysts demonstrated remarkable activity compared to literature‐reported values under similar conditions and conventional impregnated catalysts (also prepared in this work), while exhibiting high morphological stability of the exsolved NPs over prolonged operation times.

## 2 Results and discussion

### 2.1 Improving Transition‐Metal Solid Solubility in CeO_2_ via Acceptor Doping

A key challenge in the exsolution field is the low solubility limit of transition metals (e.g., Ru or Rh) in the CeO_2_ lattice, making their full incorporation difficult. To overcome this problem, which significantly hampers the extent of exsolution, a lattice modification approach was applied in this work by partial substitution of Ce^4+^ with an acceptor dopant: Gd^3+^ (Figure 1a). The rationale behind this approach can be explained through two synergistic effects: (i) lattice distortion due to the ionic radius mismatch between Gd^3+^ (1.053 Å) and Ce^4^^+^ (0.97 Å); and (ii) the formation of extrinsic oxygen vacancies ($V_{O}^{\cdot \cdot }$) associated with acceptor doping (Equation 1). As shown by Vanpoucke et al. through DFT calculations, $V_{O}^{\cdot \cdot }$ play a significant role in the lattice properties, leading to a reduction in the bulk modulus of ceria‐based materials (from 1.715 Mbar in undoped CeO_2_ to 1.588 Mbar in Gd‐doped CeO_2_) [54], thereby decreasing in lattice stiffness. Notably, dopants such as Nb and V, which do not generate oxygen vacancies (donor dopants), exhibit an inverse effect for the bulk modulus (1.871 Mbar and 1.796 Mbar, respectively) [54]. This fact supports the idea that oxygen‐vacancy‐induced lattice relaxation might facilitate the incorporation of transition metal cations such as Ru^4+^ and Rh^3+^:

(1) $$Gd_{2}O_{3}\overset{CeO_{2}}{\to }2Gd_{Ce}^{\prime }+V_{O}^{\cdot \cdot }+3O_{O}^{x}$$

Furthermore, Yao et al. successfully synthesized Ce_0.8_Sm_0.2‐x_Co_x_O_2‐δ_ without the formation of segregated cobalt oxide phases [55], despite the general understing that Co does not easily incorporate into the CeO_2_ lattice and tends to form segregated phases. This last fact can also be supported through DFT calculations, demonstrating that the defect formation energy (*E_f_*) for Co substitution in CeO_2_ is highly unfavored (*E_f_* = 11.750 eV), whereas Sm incorporation is energetically favorable (*E_f_* = −2.181 eV) [54]. Thus, the presence of Sm appeared to facilitate Co incorporation, supporting the rationale behind acceptor‐doping lattice accommodation.

In order to prove the effects of this approach in ceria‐based exsolution, three doped Ce_0.8_Gd_0.2_O_2‐δ_ materials were synthesized through the modified Pechini method (see the **Methods** section in the Supporting Information). Specifically, (Ce_0.8_Gd_0.2_)_0.94_Ru_0.06_O_2‐δ_, (Ce_0.8_Gd_0.2_)_0.94_Rh_0.06_O_2‐δ_, and (Ce_0.8_Gd_0.2_)_0.94_Ru_0.03_Rh_0.03_O_2‐δ_, mentioned hereinafter as CGORu, CGORh, and CGORuRh, respectively. These relatively high amounts of Ru and/or Rh, measured in all three materials by x‐ray fluorescence (see Table S4), were selected to assess the effect of Gd incorporation into the Ce sublattice on the solubility of Ru and/or Rh while minimizing the segregation of metal oxides. Indeed, the opposite behavior is observed for undoped Ce_0.94_Ru_0.06_O_2_, as illustrated by the slight segregation of RuO_2_ after calcination at 700 °C (PXRD analysis in Figure 1b), observable through the reflection at 2θ = 35°, since the most intense RuO_2_ reflection in the usual *P42/mnm* space group (2θ ≈ 28°) strongly overlaps with the main CGO diffraction peak. Moreover, even after sintering at higher temperatures (1100 °C) for 12 h in air (Figure S2), RuO_2_ segregations are detected, ruling out the potential positive temperature effect in the solid solubility. On the other hand, the sample with Gd^3+^ acceptor doping, CGORu, appears as a single‐phased material with no additional signals of Ru (Figure 1b). This fact was also confirmed for CGORh, and CGORuRh, in which Rh or Ru oxide segregations were not observed (Figure S3). These crystallographic results provided the first indication that the presence of Gd^3+^ in the structure is facilitating the introduction of Ru and/or Rh, thereby corroborating our design rationale. In this context, to verify the formation of $V_{O}^{\cdot \cdot }$ upon acceptor doping (Gd^3+^) and its influence on the Ru/Rh allocation, EPR analyses were performed. The obtained results comparing CGO and CeO_2_ (Figure S4a) evidence a distinct signal (*g* ≈ 2.03) associated with $V_{O}^{\cdot \cdot }$ in CGO, which is absent in undoped CeO_2_ [56, 57, 58].

Rietveld refinements showed a cubic structure, $Fm\bar{3}m$ space group for every synthesized material, exemplified for CGO in Figure S5. This sample exhibits slightly higher cell volumes (159.34 Å^3^) than the three doped CGO materials (Table 1). This fact suggests the allocation of Ru and/or Rh in Ce/Gd positions, since both Ce^4+^ and Gd^3+^ in 8‐fold coordination have larger ionic radii (0.97 and 1.053 Å, respectively) than Ru^4+^ and Rh^3+^ (0.62 and 0.665 Å, respectively, for 6‐fold coordination) [59]. DFT studies have suggested partial substitution of Ce^4+^ by Ru^4+^ in CeO_2_, leading to a lattice shrinkage [60, 61, 62], aligned with the results presented here. Additionally, as depicted in Table 1, the highest cell volume of the doped materials corresponds to CGORh (158.87 Å^3^), followed by CGORuRh‐ and CGORu samples (158.62 and 158.43 Å^3^, respectively). This fact is coherent with the Ru^4+^ and Rh^3+^ ionic radii hierarchy, indicating an effective introduction of the noble metals into the CGO lattice.

**TABLE 1:** Composition of the synthesized materials; cell volumes before (*V*_cell_) and after exsolution treatments (*V*_cell‐ex_); BET surface areas after exsolution (*S*_BET‐ex_); exsolved NPs’ mean sizes (*d_p_*) and populations; and surface metallic exsolved fractions (*ξ*_ex_).

Sample	Composition	*V*_cell_ (Å^3^)	*V*_cell‐ex_ (Å^3^)	*S*_BET‐ex_ (m^2^ g^−1^)	NP *d_p_* (nm)	NP population (NP µm^−2^)	*ξ*_ex_ (wt.%)
CGORu	(Ce_0.8_Gd_0.2_)_0.94_Ru_0.06_O_2‐δ_	158.43	160.35	12.7	3.0	2986	0.7
CGORuRh	(Ce_0.8_Gd_0.2_)_0.94_Ru_0.03_Rh_0.03_O_2‐δ_	158.62	159.98	13.7	2.5	3165	0.6
CGORh	(Ce_0.8_Gd_0.2_)_0.94_Rh_0.06_O_2‐δ_	158.87	159.76	12.3	3.4	1686	0.4
CGO	Ce_0.8_Gd_0.2_O_2‐δ_	159.34	159.40	6.5	—	—	—

### 2.2 Understanding Ru and/or Rh Exsolution in Gd‐Doped Ceria

After reduction treatments at 700 °C for 4 h under 5% H_2_/Ar flow, significant structural changes were observed. All materials exhibited cell volume expansion due to lattice chemical reduction (as evidenced by the increase in $V_{O}^{\cdot \cdot }$ concentration, observed by EPR analysis, Figure S4b), with CGORu showing the largest increase, followed by CGORuRh and CGORh, while CGO displayed the smallest, almost negligible variation (Table 1). This fact suggests alterations in the lattice due to the introduction of Ru and/or Rh cations, leading to more severe changes upon reduction compared to CGO.

**FIGURE 2:**
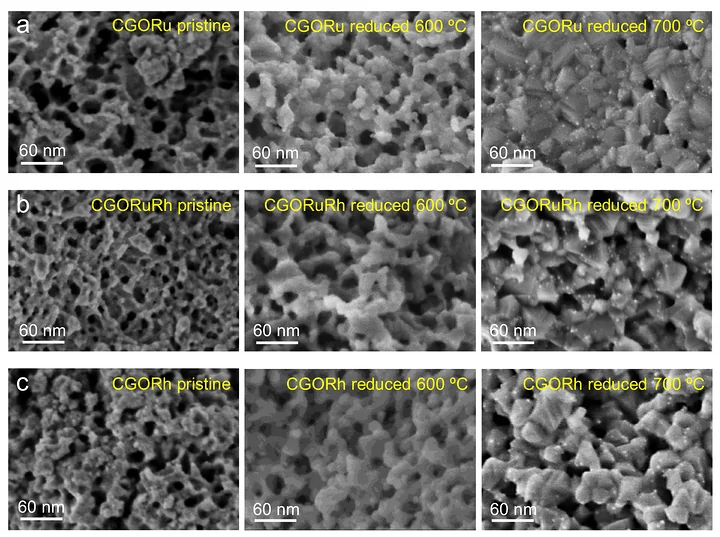
HRFESEM micrographs of the three synthesized materials, CGORu (a), CGORuRh (b), and CGORh (c). For each material, pristine, reduced at 600 °C and reduced at 700 °C (both reductions performed during 4 h under 5% H_2_/Ar flows) states are presented. Only after a 700 °C reducing treatment do exsolved nanoparticles emerge on the surfaces of the three materials.

Additionally, the narrowing of the diffraction peak widths after reduction (Figure 1c), compared with those of the as‐synthesized materials (Figure S3) indicates an increase in crystalline domain and grain sizes, following the Scherrer equation [63]. This was further corroborated by HRFESEM micrographs of pristine and reduced materials (Figure 2), which revealed notably larger grain sizes and a decrease in open porosity after reduction treatment. Importantly, reduction treatments at 700 °C induced the emergence of well‐dispersed, spherical NPs on the surfaces of the doped CGO materials, attributed to the exsolution process. It is worth mentioning that by performing the reduction at 600 °C, no sign of NP emergence was inferred (Figure 2). This indicates that (i) higher temperatures are required to efficiently trigger Ru and/or Rh exsolution from CGO, and (ii) the formation of these NPs can only be explained by Ru and/or Rh exsolution from the lattice, since reduction of Ru and/or Rh oxides occurs below 200 °C [64].

Referring to the morphological properties of the exsolved NPs (Table 1), CGORuRh exhibited the highest NP population density (3165 NPs µm^−2^), slightly surpassing CGORu (2986 NPs µm^−2^) and significantly exceeding CGORh (1686 NPs µm^−2^), suggesting higher nucleation rates for Ru compared to Rh. In terms of NP size, CGORh displayed the largest mean diameter (3.4 nm), followed by CGORu (3.0 nm) and CGORuRh (2.5 nm). However, considering the deviations in measured sizes, these differences can be considered as statistically non‐significant. The full‐size distribution histograms, along with additional details, are presented in Figure S6. The NP sizes and populations, along with the BET surface areas of the reduced materials (Table 1), were used to calculate the weight percentage (wt.%) of surface exsolved metals for each sample, ***ξ*_ex_** (Appendix S1). Across all materials, the wt.% of exsolved metals remained below 1%, as seen in Table 1. Notably, the trend in lattice expansion upon reduction is highest for CGORu, followed by CGORuRh and CGORh, and correlates with the measured quantities of exsolved metals.

**FIGURE 3:**
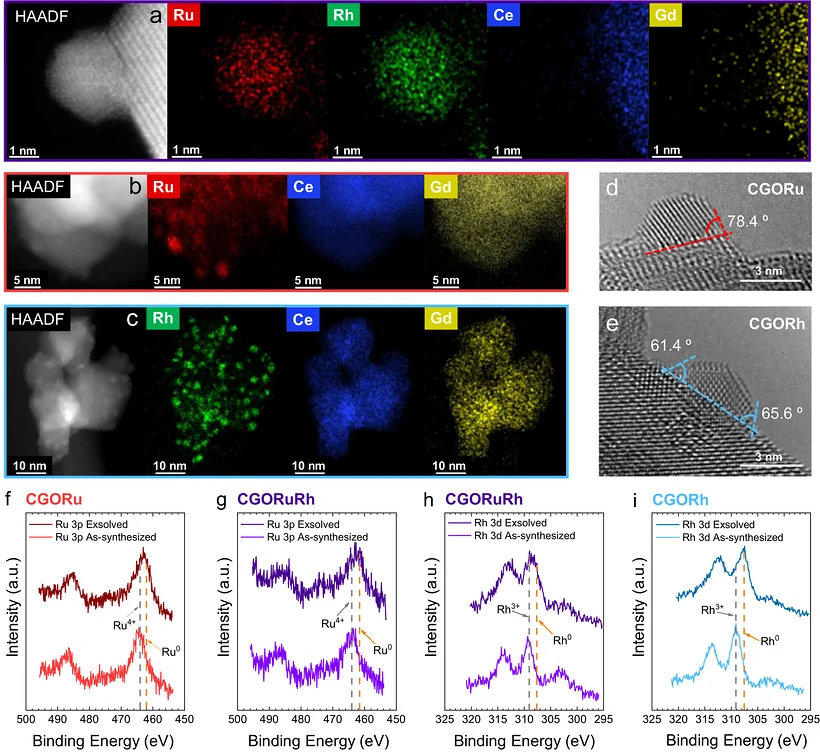
HAADF‐STEM and XEDS map images for exsolved NPs from (Ce_0.8_Gd_0.2_)_0.94_Ru_0.03_Rh_0.03_O_2‐δ_ (CGORuRh) (a). XEDS map for exsolved Ru NPs from CGORu (b). Low magnification HAADF and XEDS maps for exsolved Rh NPs from (Ce_0.8_Gd_0.2_)_0.94_Rh_0.06_O_2‐δ_ (CGORh) (c). Every exsolution was performed with reduction treatments at 700 °C for 4 h under 5% H_2_/Ar flow. HRTEM micrographs of exsolved Ru NPs from CGORu (d) and Rh NPs from CGORh (e). Contact angles between NP and support are included in both cases. XPS spectra comparison (Ru 3p and Rh 3d) between as‐synthesized and exsolved materials, analyzing the exsolvable metals from CGORu (f), CGORuRh (g), (h), and CGORh (i).

STEM and XEDS analyses were performed to exsolved materials (Figure 3a–c) as well as on the pristine CGORuRh sample to confirm the homogeneous distribution of the introduced noble metals along the lattice (Figure S7) and discard the presence of segregated Ru or Rh oxides. Additionally, the latter also exhibits consistent amounts of Ru and Rh with the results obtained by XRF. These STEM‐XEDS analyses also confirm the higher porosity in the pristine materials compared to the reduced ones, due to the sintering experienced by the materials during the exsolution treatments. Focusing on the exsolved materials, namely CGORuRh, the reduction treatment induces the exsolution of socketed, alloyed RuRh NPs (Figure 3a), marking the first report of this specific alloy exsolution in ceria‐based oxides and one of the few observed using fluorite oxides as an exsolvable matrix, alongside previously reported RuPt and RuNi systems exsolved from CeO_2_ [65, 66]. Area scan analyses (Figure S8) revealed a nearly equal metallic fraction of Ru and Rh in the NPs, with a slight enrichment in Rh. Importantly, negligible amounts of Ru and Rh were detected in the oxide matrix surrounding the exsolved NPs, indicating that these metals are predominantly extracted from the surface regions of ceria during exsolution [67, 68]. This phenomenon is well documented for other metals, such as Ni and Co [38]. Additionally, Ru and Rh exsolution was similarly confirmed for CGORu and CGORh, respectively, forming well‐dispersed NPs (Figure 3b,c, respectively).

HAADF images evidenced remarkable NP socketing (Figure 3a), potentially providing intense metal‐support interactions [69, 70]. To test this hypothesis, HRTEM analyses were conducted to characterize this socketing through the contact (wetting) angle between NPs and the support (Figure 3d,e and Figure S9). For all three exsolved materials, the vast majority of NPs exhibit contact angles below 90 °, as evidenced in CGORu (Figure 3d and Figure S9a), CGORh (Figure 3e and Figure S9d) and CGORuRh (Figure S9e). These low contact angles have been previously related to enhanced metal‐support interactions [71], especially when compared to impregnated samples, which show significantly higher than 90° NP‐support angles [72]. Additionally, the inherent partial embedment of the exsolved NPs within the host oxide surface could also be observed through HRTEM analyses (Figure S10). However, it is worth mentioning that a minor amount of the exsolved NPs emerge from pedestal‐like structures in CGORu (Figure S9b), CGORh (Figure S9c and Figure S11) and CGORuRh (Figure S12) and exhibited higher contact angles than 90° (Figure S9b,c), which might be affecting metal‐support interactions. These structures showed no detectable Ru or Rh content, suggesting that those are primarily associated with the oxide lattice and may respond to a typical support rearrangement due to suboxide formation [73], and have already been observed in other works concerning the exsolution of noble metals (Ir) [74]. Importantly, the combined characterization results suggest that both architectures originate from an exsolution‐driven process, rather than from the reduction of segregated species. Lastly, Fast Fourier Transform (FFT) patterns confirmed the presence of CGO (111) and (220) planes, with measured interplanar distances of 0.32 and 0.19 nm, respectively (Figure S13a). It is worth noting that some NPs show more complex faceted morphologies in the Rh and RuRh systems (Figure S10). However, similar features can also be observed in exsolved CGORu (Figure S13 c,d). Nonetheless, these are not the dominant morphologies overall, as most NPs are mainly spherical.

XPS further confirmed the metallic nature of the exsolved NPs (Figure 3f–i and Figure S14). In every case, after reducing treatments (700 °C, 4 h, and 5% H_2_/Ar flow), metallic species of Ru and/or Rh emerged, due to the formation of exsolved NPs. Referring to the Ru‐containing materials (Figure 3f,g), the oxide component corresponding to Ru^4+^ is located around 464 eV, whereas after exsolution, a predominant component at 462 eV corresponding to metallic Ru (Ru 3p_3/2_) [75] appeared, confirming the exsolution of Ru species. It is important to mention that the Ru 3p core shell was used to identify the exsolution process, as Ru 3d overlaps with C 1s, resulting in specific difficulties for the analyses [76]. Similarly, in Rh‐containing materials (Figure 3h,i), after the reduction treatment, a clear shift to lower binding energies could be appreciated, indicating the reduction of Rh^3+^ into metallic Rh. Namely, oxidized Rh^3+^ species contained in the CGO lattice correspond to a peak at 309 eV (Rh 3d_5/2_), whereas after exsolution, the main component is centered at around 307 eV. This shift was more pronounced for the CGORh than for CGORuRh, as the latter exhibits NP with lower Rh contents. CGORh spectra for the other components (O 1s, Ce 3d, and Gd 3d), as well as the deconvolution of Rh 3d, are shown in Figure S14. Ce 3d deconvolution showed the presence of both Ce^4+^ and Ce^3+^ species. Rh 3d deconvolution confirmed that, after exsolution, Rh^3+^ remained in the oxide bulk, but to a lesser extent than Rh exsolutions.

**FIGURE 4:**
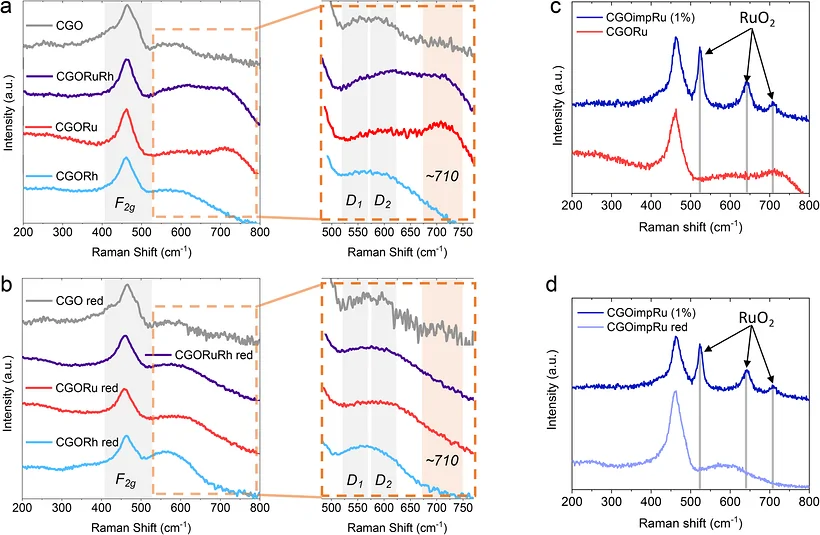
Raman spectra (logarithmic scale) of the synthesized materials in the pristine state (a) and after reduction at 700 °C, 4 h under 5% H_2_/Ar flow (b). For both states, a zoom‐in of the 550–750 cm^−1^ Raman shift is provided. Raman spectra comparison between CGOimpRu (Ru = 1 wt.%) and CGORu, which reveals the absence of active modes of RuO_2_ in CGORu (528, 644, and 716 cm^−1^; *E_g_*, *A_1g_*, and *B_2g_*, respectively). Additionally, the Gd‐O‐Ru bonding mode (∼710 cm^−1^) cannot be seen in CGOimpRu (c). Raman spectra comparison between pristine and reduced (red) CGOimpRu (d). After reduction treatment, the Raman‐active modes of RuO_2_ are no longer detectable.

Raman spectroscopy was carried out on both pristine and exsolved materials to investigate additional structural features. For the pristine samples (Figure 4a), the most intense Raman‐active mode, observed at ∼460 cm^−1^, corresponds to the *F_2g_* mode of cubic $Fm\bar{3}m$ CeO_2_ [48, 77]_._ An asymmetrical broadening at higher wavenumbers (∼480 cm^−1^) is attributed to Ce partial substitution with Gd [77]. This broadening, commonly observed when introducing trivalent cations into CeO_2_, arises from the formation of non‐equivalent bonds within the fluorite lattice [77, 78]_._ Although not resolved as a distinct vibrational mode, this feature confirms the incorporation of Gd into the oxide lattice.

In all samples, both pristine and reduced, overlapping modes at ∼550 cm^−1^ (*D_1_*) and ∼600 cm^−1^ (*D_2_*) were observed, which are absent in pure CeO_2_ [77]_._ These modes are widely associated with oxygen vacancies created by Gd^3+^ incorporation [79, 80]. While the 600 cm^−1^ mode has also been linked to lattice defects involving the dopant, the 550 cm^− 1^ mode is more specifically attributed to oxygen vacancy defects [81]. An additional mode at ∼710 cm^−1^ was identified in CGORu and CGORuRh but was absent in CGO and CGORh, suggesting its origin lies in the presence of Ru within the lattice. This mode is consistent with a signal observed at ∼690 cm^−1^ in Sm_2_Ru_2_O_7_, which was attributed to Sm‐O‐Ru bonding [47]. Here, the shift to ∼710 cm^−1^ can be attributed to Gd‐O‐Ru bonding, which differs due to the substitution of Gd for Sm. The absence of RuO_2_‐related modes (528, 644 and 716 cm^−1^; *E_g_*, *A_1g,_* and *B_2g_*, respectively) further supports the incorporation of Ru into the lattice rather than forming secondary phases [47, 82].

In contrast, CGORh did not exhibit the ∼710 cm^−1^ mode, indicating that the Gd‐O‐Rh environment differs from Gd‐O‐Ru. This is reasonable given the different oxidation states of Ru^4+^ and Rh^3+^ in the CGO lattice. In other studies of Rh‐doped CeO_2_, a broad Raman signal overlapping with *D_1_* and *D_2_* modes (∼550–600 cm^−1^) was observed across calcination temperatures, which could explain the lack of additional modes in CGORh [83]. The absence of Rh_2_O_3_ signals (276 and 422 cm^−1^) in CGORh and CGORuRh further confirms the incorporation of Rh into the lattice, consistent with PXRD data (Figure S3) [84].

Upon reduction (Figure 4b), the ∼710 cm^−1^ mode disappeared in CGORu and CGORuRh, indicating the migration of Ru cations from the lattice to the surface due to the exsolution of metallic Ru NPs. For CGORh, the narrowing of the 550–600 cm^−1^ modes suggests a similar alteration in the Gd‐O‐Rh environment due to Rh exsolution into metallic NPs. All reduced materials showed a shift of the *F_2g_* mode to lower wavenumbers (Table S5), consistent with lattice expansion caused by chemical reduction [48, 77, 85], as also confirmed by PXRD results (Table 1).

To corroborate these conclusions, a 1 wt.% Ru‐impregnated CGO material (CGOimpRu) was also synthesized (see the **Methods** section) for establishing a Raman spectra comparison with the exsolved sample, CGORu (Figure 4c). In the former, RuO_2_ typical modes (528, 644, and 716 cm^−1^) appear but are absent in the latter. Furthermore, after reduction of CGOimpRu, these RuO_2_ modes are no longer appreciable (Figure 4d), consistent with the formation of impregnated Ru metal species. Additionally, PXRD analyses of the pristine CGOimpRu do not show any RuO_2_ signal (Figure S15), just as the case for CGORu (Figure 1b). Further, the reduction process evidences a scarce, non‐significant cell volume expansion after 700 °C, 4 h treatments (from 159.50 to 159.54 Å^3^, Figure S15), in line with the behavior observed for undoped CGO (Table 1). This fact confirms that the larger lattice expansions observed upon reduction of the doped materials imply alternative lattice alterations due to exsolution of Ru and/or Rh (Table 1). Additionally, HRFESEM micrographs of pristine and 700 °C‐reduced CGOimpRu (Figure S16) reveal morphological differences relative to CGORu and the absence of small nanoparticles after the reduction treatment, as observed in CGORu due to exsolution (Figure 2a).

Building on the above experimental evidence confirming NP exsolution, although it has been extensively described in perovskite hosts, the process can be rationalized within a defect‐chemistry framework adapted to fluorite oxides. The formation and growth of exsolved NPs in CGO can be understood through $V_{O}^{\cdot \cdot }$ formation, electron generation, and dopant reduction driving metal segregation and nucleation. In Ce_0.8_Gd_0.2_O_2‐δ_, aliovalent substitution of Ce^4+^ with Gd^3+^ introduces an increased concentration of $V_{O}^{\cdot \cdot }$ (as demonstrated by EPR analyses), generating a defect‐rich lattice with enhanced oxide‐ion mobility. Alternatively, considering Ru^4+^ incorporation into the lattice (confirmed by PXRD and Raman) as an explanatory model and assuming isovalent substitution of Ce^4+^, major charge compensation would not be required. Under reducing conditions, the number of $V_{O}^{\cdot \cdot }$ (which, in addition, act as preferential nucleation sites for exsolution) is further increased via lattice oxygen removal, accompanied by polaron formation and electron transfer. The reduction of incorporated Ru species is then facilitated by these electrons, enabling migration and NP formation at the surface of Ru^0^ (as observed by HRFESEM, STEM, and XPS), generating cation vacancies. In addition, these electrons could participate in the redox buffering reaction of the Ce cations. The intrinsically oxygen‐deficient nature of Gd‐doped ceria provides a favorable environment for exsolution, where the interplay between $V_{O}^{\cdot \cdot }$ concentration, Ce^4+^/Ce^3+^ redox buffering, and dopant reduction governs the extent and kinetics of Ru NP formation.

In summary, this section has provided evidence of metallic NP exsolution from Gd‐doped ceria. This lattice‐tuning strategy via acceptor doping has been shown to enhance Rh^3+^ and Ru^4+^ incorporation in the fluorite lattice, thereby preventing RuO_2_ or Rh_2_O_3_ segregation. Structurally, both acceptor doping and reduction treatments generated oxygen vacancies in the catalyst support, extrinsic and intrinsic, respectively. This is a key aspect of the exsolution process, since the generation of oxygen vacancies is a prerequisite for the nucleation of catalytically active exsolved metallic nanoparticles at the oxide surface [86].

### 2.3 Catalytic Tests for H_2_ Production via NH_3_ Decomposition

**FIGURE 5:**
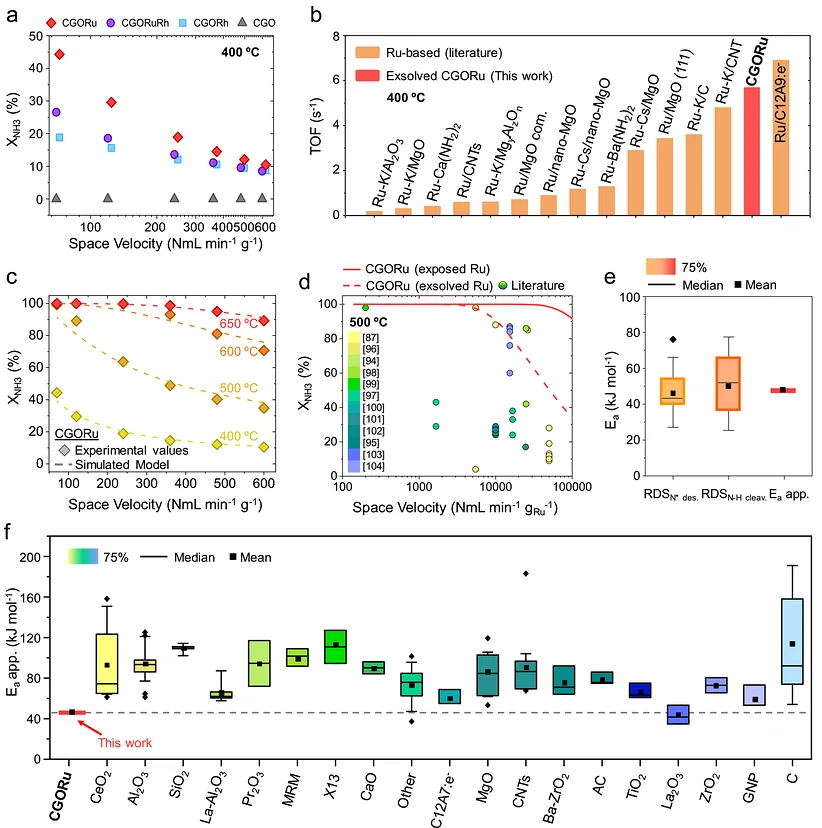
NH_3_ decomposition conversion as a function of the space velocity at 400 °C for CGO, CGORu, CGORuRh, and CGORh exsolved catalysts (a). Comparison of experimental and model values for the CGORu exsolved catalyst at every tested temperature (b). TOF comparison of exsolved CGORu (this work) with different catalysts reported in the literature for NH_3_ decomposition reaction at 400 °C, Ru‐based [9, 87, 88, 89, 90, 91, 92, 93] catalysts. Similar or higher space velocities to 600 NmL min^−1^ g^−1^ (approximately 36 000 mL h^−1^ g^−1^) were considered (c). X_NH3_ as a function of the Ru‐based space velocity, considering the Ru mass both the exposed and exsolved, for CGORu at 500 °C, framed in a comparison with different literature results (500 °C) [87, 94, 95, 96, 97, 98, 99, 100, 101, 102, 103, 104] (d). Comparison of the calculated *E_a_* for exsolved CGORu through the proposed model, considering two different rate‐determining steps: RDS_N* des._, RDS_N‐H cleav._, and calculated through experimental values, apparent *E*_a_. The boxes represent 75% of the simulated values, and the whiskers are calculated as 1.5IQR. Outliers are represented as rhombi (e). Comparison of the *E*_a_ app. of exsolved CGORu with literature works dealing with different Ru‐based catalysts [21, 87, 90, 92, 93, 94, 95, 98, 102, 105, 106, 107, 108, 109, 110, 111, 112, 113, 114, 115, 116, 117, 118, 119, 120, 121, 122, 123, 124, 125, 126, 127, 128, 129, 130, 131, 132, 133, 134, 135, 136, 137, 138, 139, 140, 141, 142] (f).

The catalytic performance of the exsolved materials for NH_3_ decomposition was systematically evaluated in a fixed‐bed reactor. Each catalyst underwent in situ exsolution before testing at varying temperatures and space velocities, as detailed in the **Methods** section (Table S1). NH_3_ conversion results under different reaction conditions are presented in Figure 5a (400 °C) and Figure S17 (650 °C, 600 °C, and 500 °C). Every represented conversion value remained stable across all tested conditions, as exemplified for CGORu in Figure S18. For comparison, in situ reduced CGO was tested under identical conditions. As a general trend, NH_3_ conversion increased with higher temperatures and lower space velocities across all catalysts. Importantly, every exsolved material significantly outperformed CGO, underscoring the critical role of Ru and/or Rh exsolution in enhancing catalytic performance. CGO exhibited negligible catalytic activity, particularly at lower temperatures (500 °C and 400 °C). At higher temperatures (600 °C and 650 °C), CGO performance improved slightly, mainly due to NH_3_ thermal decomposition, but remained far behind that of the doped materials.

Focusing on these doped catalysts, exsolved CGORu consistently exhibited the highest catalytic performance, followed by CGORuRh and CGORh. This establishes a clear correlation between Ru content and NH_3_ conversion, aligning with findings by Ganley et al. [16], who reported a catalytic activity hierarchy of Ru > Ni > Rh. At lower temperatures, where thermal decomposition effects are less pronounced, these differences in catalytic performance become more evident. For example, at 500 °C and a low space velocity (∼70 NmL min^−1^ g^−1^), CGORu achieved complete NH_3_ conversion (100%), whereas CGORuRh and CGORh reached only ∼77% and ∼65%, respectively (Figure S17). At 400 °C (Figure 5a), the same catalytic performance hierarchy was observed, with differences becoming most pronounced at lower space velocities. Specifically, at ∼70 NmL min^−1^ g^−1^, CGORu achieved an NH_3_ conversion of 45%, compared to 26% for CGORuRh and 19% for CGORh. HRFESEM post‐mortem analyses were performed for all materials after the reaction tests (Figure S19), confirming that exsolved NPs remain stable along the surface of every catalyst.

Turnover frequencies (TOFs) were calculated for each exsolved catalyst at 400 °C and 600 NmL min^−1^ g^−1^ (Appendix S2). This space velocity and temperature were selected to ensure low NH_3_ conversion (approximately 10%), thereby avoiding equilibrium effects and compositional variations due to the reaction process. The available catalytic sites for NH_3_ decomposition were estimated assuming that the surface‐exposed metal fractions (thus, the embedded NP fraction in the host oxide surface is not considered as available active site) are hemispherical nanoparticles, as detailed in Appendix S2. This estimation is based on previous TOF calculations for exsolved catalysts based on indirect methods [68]. As a result, the TOF values obtained were: 5.7 s^−1^ for CGORu; 5.9 s^−1^ for CGORuRh; and 6.4 s^−1^ for CGORh. Focusing on exsolved CGORu (the best‐performing catalyst in terms of NH_3_ conversion), a TOF comparison with other Ru‐based catalysts reported in the literature (Table S6) is depicted in Figure 5b. This comparison considered only 400 °C and similar or higher space velocities to 600 NmL min^−1^ g^−1^ (36,000 NmL h^−1^ g^−1^) studies. Under these conditions, exsolved CGORu (also CGORu and CGORuRh) showed high performance in TOF terms compared with previously reported Ru‐based catalysts for NH_3_ decomposition [9, 87, 88, 89, 90, 91, 92, 93], illustrating the remarkable catalytic potential of these materials and highlighting the benefits of the exsolution process.

### 2.4 Kinetic Modeling for H_2_ Production via NH_3_ Decomposition

The hydrogen production rate of the tested materials was analyzed using kinetic modeling (see Appendix S3 and Tables S7–S9 for more details), considering three different approaches based on Sayas et al. work [121]. The best model fitting (Figure S20) was obtained considering the first N‐H cleavage as rate‐determining step (RDS_N‐H cleav._) and showed excellent agreement with experimental results across all materials and temperature ranges (Figure 5c and Figure S21). Activation energies (*E_a_*) were calculated through the three different simulation approaches (Tables S7–S9) and compared to the apparent *E_a_* (*E_a_* app.) obtained with the experimental results. Focusing on exsolved CGORu, *E_a_* app. (∼50 kJ mol^−1^) shows very similar values to calculated RDS_N‐H cleav._ and RDS_N* des._ (desorption of adsorbed nitrogen) activation energies (50.9 and 39.6 kJ mol^−1^, respectively), Figure 5e, and are among the lowest *E_a_* values reported in Ru‐based catalysts for NH_3_ decomposition reactions [9]. This fact is well illustrated in Figure 5f, which compares the *E_a_* app. for CGORu (this work) with values reported in the literature for catalysts evaluated using the same methodology (*E_a_* app.).

Based on the simulated model and focusing on exsolved CGORu, an additional comparison with the literature is shown in Figure 5d. The Ru‐based space velocity was defined based on the Ru mass (instead of the catalyst mass). The figure shows the results at 500 °C for both the literature values and this work as a function of the Ru‐based space velocity. For the results of this work, both the overall Ru exsolved (*ξ_ex_* = 0.7%) and the exposed Ru (6.5·10^−6^ mol_Ru_ g^−1^_cat_) have been used. These results show that the exsolved surface‐available metallic Ru (thus, catalytically active) presents apparently higher activity than the impregnated‐based catalysts in the literature, especially at higher temperatures (see Figure S22, 400 °C). Here, it is worth noting that the intrinsic properties of exsolution will play a key role in improving catalytic performance. Although the main active sites for NH_3_ decomposition (i.e., initial N‐H bond cleavage) are generally associated with exposed metallic Ru^0^ sites [143], there is growing evidence that they are not the only determining catalytic factor. The Ru‐CeO_2_ interface plays a significant role in modulating catalytic performance by (i) increasing the electron density of the exposed Ru NPs through strong metal‐support interactions [105] and (ii) involving specific interfacial configurations associated with oxygen vacancies (e.g., Ru‐$V_{O}^{\cdot \cdot }$ ‐Ce environment), which can further promote electronic redistribution effects [144]. Therefore, both contributions may synergistically enhance NH_3_ decomposition.

To further explore this aspect, additional NH_3_ Temperature Programmed Desorption (NH_3_‐TPD) and CO chemisorption experiments were performed on exsolved CGORu, exsolved CGORuRh, and reduced CGOimpRu (1 wt.% Ru) (Figure S23). NH_3_‐TPD analyses (Figure S23a) revealed that H_2_ formation from adsorbed NH_3_ starts at lower temperatures for the exsolved catalysts than for the impregnated counterpart, which suggests an enhanced catalytic activity [145, 146] and thus indicates that the exsolution‐derived architecture facilitates NH_3_ decomposition. Furthermore, CO chemisorption showed that exsolved CGORu exhibits a substantially larger active area per gram of metal than CGOimpRu (109.4 vs. 76.2 m^2^ g^−1^), despite containing a lower estimated amount of surface Ru. This result confirms a more efficient utilization and dispersion of the active metallic phase through exsolution.

**FIGURE 6:**
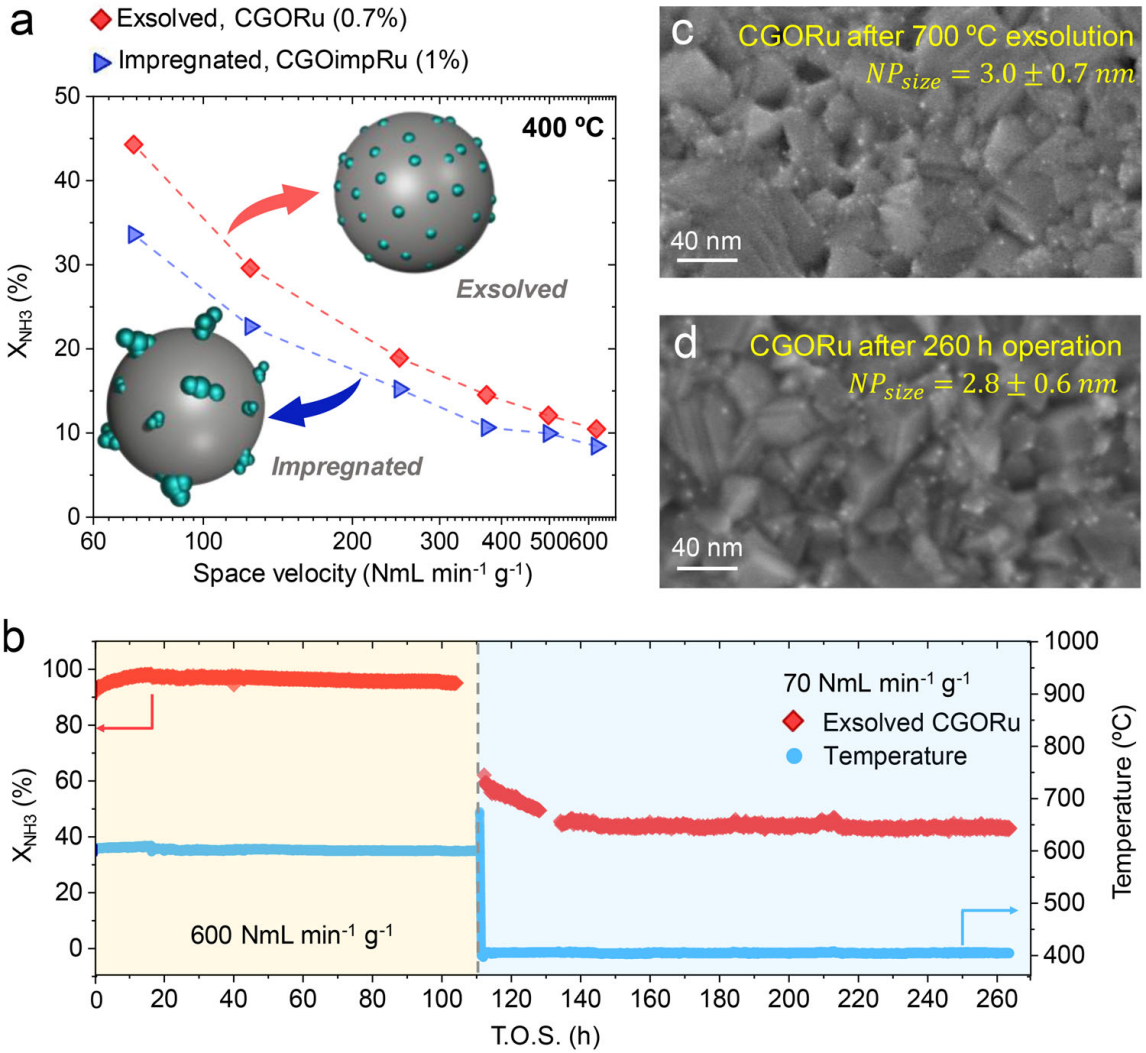
NH_3_ decomposition conversion comparison between exsolved CGORu and in situ reduced CGOimpRu at 400 °C as a function of space velocity (a). 260 h long‐term stability tests for exsolved CGORu at two different temperatures: first, at 600 °C (110 h) and second, 400 °C (150 h) (b). HRFESEM micrographs of exsolved CGORu before (c) and after long‐term stability tests (d), including NP mean sizes.

Interestingly, exsolved CGORuRh also exhibited a significantly higher active area per gram of metal (101.0 m^2^ g^−1^), yet its catalytic performance remained lower than for both exsolved CGORu and CGOimpRu (see Figures 5a and 6a). This observation indicates that catalytic activity cannot be directly correlated with the density of exsolution‐induced interfacial sites alone and should not be considered the primary catalytic active sites. Instead, the combined catalytic, NH_3_‐TPD, and chemisorption results suggest that exposed metallic Ru remains the primary active phase governing NH_3_ decomposition, where exsolution definitely contributes by enhancing the accessibility, utilization, and catalytic efficiency of these Ru sites compared to impregnation. Therefore, the improved performance of CGORu is mainly attributed to the effect of highly dispersed, accessible Ru species, with an important contribution from the beneficial metal‐support interactions generated during exsolution (as TPD analyses demonstrate), rather than to the Ru‐CGO interface acting as an independent, dominant active site.

Although the exsolved NPs exhibit remarkably high activities, the system requires further optimization to increase the exsolved metallic fraction and the BET surface area, which in turn will further increase the catalytic activity. For instance, by improving the textural properties of the host oxide support, for example, by reducing the ceria crystallite and, therefore, minimizing the Ru fraction retained in the grain interior. In this sense, calculations in Appendix S4 indicate that using highly porous supports could increase the exsolution surface area, thereby improving catalytic performance.

### 2.5 Exsolution Versus Impregnation and Long‐Term Stability Tests

To prove the advantages of exsolution versus impregnation, exsolved CGORu was benchmarked against the impregnated Ru catalyst prepared (CGOimpRu, 1 wt.% Ru; *S*_BET‐red_ = 10.3 m^2^ g^−1^), intentionally selected to slightly exceed the estimated exsolved Ru content to account for the inherent uncertainty associated with NP population and size measurements. Exsolved CGORu outperformed CGOimpRu across all space velocities at 400 °C, selected to minimize thermal decomposition effects (Figure S24), with the differences in conversion being more evident at lower space velocities (Figure 6a). For example, at ∼70 NmL min^−1^ g^−1^ space velocity, CGORu achieved a 45% NH_3_ conversion, compared to 32% for CGOimpRu, despite a lower metallic Ru surface fraction (0.7% for CGORu vs. 1% for CGOimpRu). This comparison highlights the impact over catalytic activity that the exsolution process can provide, ascribed to synergistic effects of inherently anchored, highly homogeneous, small‐sized NPs, and better distribution of the metallic active phase. However, further optimization aimed at maximizing the fraction of exsolved active species represents a key direction for improving overall catalyst efficiency.

To prove the morphological stability of the exsolved NPs, long‐term catalytic tests of the exsolved CGORu sample were carried out (Figure 6b). For these experiments, two consecutive temperatures were evaluated: 600 °C for ∼110 h and 400 °C for ∼150 h. These tests guarantee a proper evaluation of the exsolved NPs at the most interesting temperature to avoid thermal cracking (400 °C) after being submitted to harder conditions (600 °C), which might cause catalyst deactivation due to NPs’ coarsening or growth. Nonetheless, after the initial ∼110 h at 600 °C, the catalyst exhibited excellent stability and reproducibility at 400 °C during ∼150 h. Moreover, HRFESEM micrographs comparing fresh ex situ exsolved CGORu (Figure 6c) and post‐mortem analyses after 260 h of reaction performed by HRFESEM (Figure 6d) and HRTEM (Figure S25) revealed the absence of degradation. Exsolved NPs remain well‐dispersed and show no signs of growth or coarsening, retaining NP sizes within the experimental uncertainty of both HRFESEM and HRTEM, with no statistically significant differences compared to the exsolved catalyst before the long‐term test (Figure S26). Referring to the support, no signs of degradation were observed (Figure S27). In addition, the post‐mortem XRD analysis after 260 h operation shows no detectable lattice expansion (i.e., no shift towards lower 2*θ* values), indicating that no further reduction of Ce^4+^ to Ce^3+^ takes place under reaction conditions. Consistently, no increase in NP population was observed after long‐term testing, suggesting that no additional nucleation events occur. Taken together, these results indicate that the concentration of $V_{O}^{\cdot \cdot }$ remains essentially unchanged during NH_3_ decomposition, and thus, further vacancy formation under reaction conditions can be reasonably excluded. These facts highlight the remarkable stability and robustness of these catalysts based on exsolved NPs, as well as the key role of the strong anchoring of exsolution‐derived NPs to the support.

In summary, exsolved CGORu exhibited excellent catalytic activity compared to conventional impregnated CGOimpRu with lower metallic Ru available amounts, consistently outperforming the latter at every space velocity and for 72 h on stream at 400 °C and 70 NmL min^−1^ g^−1^. Additionally, the robustness of the exsolved CGORu catalyst was demonstrated through a 260 h long‐term catalytic test, including two different temperatures. The exsolved NPs demonstrated excellent catalytic activity, stability, and durability, providing a remarkably powerful catalyst design strategy for high‐interest chemical processes, such as NH_3_ decomposition for H_2_ production.

## 3 Conclusion

The effective dissolution of Ru and/or Rh transition metals into the Ce_0.8_Gd_0.2_O_2‐δ_ lattice proved that modulation of the cerium oxide structure enhances the partial substitution of Ce cations for exsolvable metals. The incorporation of an acceptor dopant (Gd^3+^) is a determining factor for the proper stabilization of the introduced cations due to a lattice flexibility enhancement. This effect is caused by the combination of lattice distortion due to the size mismatch between Ce^4+^ and Gd^3+^ and the formation of extrinsic oxygen vacancies. The latter leads to a decrease in the bulk modulus (as shown in DFT literature calculations), which lowers the lattice stiffness, enabling a better allocation of smaller metal transition cation dopants. This allows the introduction of higher amounts of exsolvable species compared to previous reports on CeO_2_‐based systems, which potentially leads to increased availability of transition metals for NP promotion through exsolution. The absence of RuO_2_ and Rh_2_O_3_ segregations was demonstrated at the long range with PXRD, morphologically with STEM, HRFESEM, and at the near order with Raman spectroscopy.

Thermal reduction enabled the exsolution of highly dispersed, homogeneously sized (2–3 nm) metallic nanoparticles, including RuRh alloys, in fluorite‐type materials (reported here for the first time). HRFESEM analyses revealed a greater extent of exsolution for Ru than for Rh. The exsolved catalysts exhibited remarkable performance for NH_3_ decomposition, with CGORu consistently outperforming CGORuRh and CGORh across all tested temperatures (400–650 °C). This superior activity correlated with the higher metallic Ru content in the exsolved nanoparticles. Notably, CGORu exhibited excellent stability during prolonged operation (260 h) at 400 °C after being exposed to 600 °C, maintaining high NH_3_ conversion rates without signs of degradation affecting the NPs.

Compared to conventional impregnation methods, exsolution proved advantageous, as CGORu achieved higher NH_3_ conversion rates than an impregnated catalyst, despite a lower metallic Ru surface content (0.7 vs. 1 wt.%, respectively). Turnover frequency (TOF) calculations further highlight the remarkable intrinsic catalytic activity of the exsolved materials, comparable to some of the best‐performing state‐of‐the‐art catalysts tested under comparable conditions. Surface oxygen vacancies in Ru‐depleted CGO host promote strong interactions between exsolved nanoparticles and support, modulating the electronic structure of Ru, facilitating electron transfer, and enhancing NH_3_ dissociation kinetics, as demonstrated through kinetic simulations performed and the *E_a_* comparisons established with other reported catalysts. Collectively, these structural and electronic effects synergistically enhance activity while providing sustained stability in NH_3_ decomposition. Overall, this work highlights the importance of rational oxide lattice design to enhance the exsolution process, offering a scalable route and confirming that structure‐performance relationships are critical for designing next‐generation catalysts for efficient hydrogen production via NH_3_ decomposition. These findings contribute to extending the exsolution process to this distinct oxide family, offering new design principles for cation incorporation and nanoparticle exsolution in ceria‐based systems. Moreover, this work establishes exsolution‐based CeO_2_ materials as a promising platform for sustainable energy applications.

## Author Contributions

**Andrés López‐García**: conceptualization, experimental investigation, data analysis, writing – original draft. **Elena Barrio‐Querol**: experimental investigation, data analysis. **Álvaro Represa**: conceptualization, experimental investigation, writing – review. **Jesús Ara**: investigation, data analysis. **Ana B. Hungría**: experimental investigation, data analysis. **David Catalán‐Martínez**: investigation, data analysis, kinetic modelings, writing – review and editing. **Alfonso J. Carrillo**: conceptualization, supervision, data analysis, project administration, funding, writing – review and editing. **Sonia Escolástico**: conceptualization, data analysis, supervision, project administration, funding, writing – review and editing. **José Manuel Serra**: conceptualization, supervision, project administration, funding, writing – review and editing.

## Conflicts of Interest

The authors declare no conflicts of interest.

## Supporting information




**Supporting File**: anie73784‐sup‐0001‐SuppMat.pdf.

## Data Availability

The data that support the findings of this study are available from the corresponding author upon reasonable request.
