# Supplementary material for: Acceptor‐Doped Cerium Oxides Enabling Metallic Nanocatalyst Exsolution for Hydrogen Production From Ammonia
Source: Angew Chem Int Ed Engl. 2026 Jul 24;65(39):e6182981. doi: 10.1002/anie.6182981 (PMC13592327; doi:10.1002/anie.6182981)
Supplement: Supplementary file 1 [file ANIE-65-e6182981-s001.pdf]

Supporting information for

Acceptor-doped cerium oxides enabling metallic nanocatalyst exsolution for hydrogen production from ammonia

Andrés López-García[1], Elena Barrio-Querol[1], Álvaro Represa[1], Jesús Ara[1], Ana B. Hungría[2], David Catalán-Martínez[1], Alfonso J. Carrillo[1*], Sonia Escolástico[1*], José Manuel Serra[1*]

[1]Instituto de Tecnología Química (Universitat Politècnica de València-Consejo Superior de Investigaciones Científicas), 46022 València, Spain.

[2]Departamento de Ciencia de Materiales, Ingeniería Metalúrgica y Química Inorgánica, Universidad de Cádiz, Campus Río San Pedro, 11510 Puerto Real, Spain.

Corresponding authors: soesro@itq.upv.es; alcardel@itq.upv.es; jmserra@itq.upv.es

Methods

Material synthesis

$Ce_{0.8}Gd_{0.2}O_{2-\delta}$ (CGO) and doped $(Ce_{0.8}Gd_{0.2})_{0.94}M_{0.06}O_{2-\delta}$ materials (with M = Ru, Rh and RuRh) were synthesized via modified sol-gel Pechini method. Metal precursors, namely $Ce(NO_3)_3{\cdot}6H_2O$ (99.5%, Alfa-Aesar), $Gd(NO_3)_3{\cdot}6H_2O$ (99.9%, ABCR), $RuHN_4O_{10}$ (Ru=1.5 wt.%, Aldrich) and $Rh(NO_3)_3{\cdot}xH_2O$ (Rh=36 wt.%, Aldrich) in appropriate amounts were introduced and dissolved in 100 mL deionized water at 60 ºC under 250 rpm constant stirring. For this purpose, citric acid (CA) was added as a complexing agent (99%, ThermoScientific) in a molar ratio 1:1.5 (metal precursor:CA). After every precursor is dissolved, ethylene glycol (EG, 99%, Aldrich) was introduced in the dissolution (CA/EG ratio of 2/3 wt. %) as the organic component for the gelification. After this, the temperature was increased to 80 ºC to eliminate water before transferring the dissolution to a drying oven (220 ºC, overnight) to induce gelification and further calcination. The obtained solid was ground to a fine powder in an agate mortar, sieved through a 200 μm sieve, and calcined at 700 ºC for 5 h. This temperature ensured the formation of single-phase materials, along with some porosity and, thereby, higher surface areas. Reduction treatments for exsolution were carried out in a quartz horizontal reactor at 700 ºC for 4 h, under 5% H_2/Ar flow.

To compare catalytic performance between exsolution and impregnation, 1%-Ru-impregnated CGO (CGOimpRu) was prepared by the incipient wetness method. The synthesized CGO was impregnated with appropriate amounts of the Ru precursor and heated to 600 ºC for 5 h.

Physicochemical characterization

Powder X-Ray Diffraction (PXRD) analyses were performed with a PANalytical CubiX diffractometer with Cu $K\alpha 1,2$ radiation and a Bragg-Brentano X'Celerator detector. Rietveld refinements of the synthesized structures were made with X'Pert HighScore Plus (version 3.0.0).

High-Resolution Field Emission Scanning Electron Microscopy (HRFESEM) was used to examine the surfaces of the synthesized materials before and after exsolution

treatments. For this, a GeminiSEM 500 electron microscope from Zeiss Oxford Instruments was used. The size and population of the exsolved NPs were studied using ImageJ (version 1.52a). For High-Resolution Transmission Electron Microscopy (HRTEM), a double aberration-corrected FEI Titan^3 Themis 60-300 microscope was employed. Analyses were carried out at 200 kV. This instrument, equipped with a monochromatic XFEG source and a Super-X-EDS system (consisting of four symmetrically disposed windowless SSD detectors), was also employed for Energy-Dispersive X-ray Spectroscopy (EDX) and for Scanning Transmission Electron microscopy (STEM). For XEDS measurements, the beam current was set to 50 pA, the convergence angle to 19 mrad, and the 20 keV range.

Raman spectroscopy was performed using a Renishaw Raman spectrometer, at room temperature, using a 785 nm laser equipped with an Olympus microscope and a CCD detector.

BET surface area was obtained for each material after reduction treatment (700 ºC, 4 h, 5% H_2/Ar flow) using a Micrometrics ASAP2420 equipment. The adsorptive gas employed was N_2, and the bath temperature was -196 ºC. In every case, between 0.20 and 0.25 g of material was analyzed.

X-ray photoelectron spectroscopy (XPS) was used to study the composition of the exsolved NPs. These analyses were performed with a SPECs spectrometer, using an Al-Kα monochromatic source and an MCD-9 detector. The spectra were calibrated with the adventitious C1s signal at 284.8 eV.

EPR spectra were measured at room temperature in a Bruker EMX 10/12 instrument operating at X-Band, using a modulation frequency of 100kHz and a modulation amplitude of 1G. The g-factor associated with oxygen vacancies was determined from the resonance field (inflection point of the signal, g ≈ 2.03), calculated through[1–3]:

$$g = \frac{h\nu}{\beta H} \qquad \textbf{(Eq. S1)}$$

Where $\boldsymbol{h}$ is the Planck constant, $\boldsymbol{\nu} \approx 9.85$ GHz; $\boldsymbol{\beta}$ the Bohr magneton constant; and $\boldsymbol{H}$ the magnetic field at which the signal is observed[1].

X-Ray Fluorescence (XRF) measurements were carried out at room temperature using a Zetium spectrometer (Malvern Panalytical, 4 kV) equipped with an Rh-anode X-ray source. Powder samples were analyzed under a He atmosphere.

Ammonia Temperature-Programmed Desorption (NH3-TPD) experiments were performed in a quartz tubular reactor for exsolved CGORu, CGORuRh, and reduced CGOimpRu (1% Ru). The pre-exsolved (or reduced) materials were submitted to NH_3 flows at 50 ºC during 1 h for ammonia adsorption. After that, desorption was performed under Ar flow at a heating rate of 9 ºC min^{-1} to 700 ºC. During the desorption step, the evolution of H_2 and N_2 was continuously monitored using a mass spectrometer.

CO chemisorption measurements for exsolved CGORu, exsolved CGORuRh and CGOimpRu (1% Ru) were carried out using an Anton Paar Autoabsorb 6200 FKM MP instrument. The analysis temperature was set at 35 ºC, a CO:M stoichiometry of 1:1 was considered, and between 0.3-0.4 g of each sample was employed. Surface areas per gram of metal were obtained by extrapolating the total gas uptakes in the adsorption isotherms at P = 0 torr.

Catalytic tests

NH_3 decomposition catalytic tests were carried out in a quartz fixed-bed tubular reactor (inner diameter of 0.86 cm). The employed mass of each catalyst was 0.500 g. Every catalyst was first *in-situ* treated under 5% H_2/Ar flow for 5 h for exsolution of metallic NPs. The NH_3 conversion was studied as a function of different parameters, as detailed in **Table S1**.

**Temperature (ºC)**	650-600-500-400						
**Flow rate (mL min^{-1})**	**NH_3**	25	50	100	150	200	250
	**He**	10	10	20	30	40	50
**He percentage (%)**		28.6	16.7	16.7	16.7	16.7	16.7
**Space Velocity (NmL min^{-1} g^{-1})**		70	120	240	360	480	600

Table S1. Experimental parameters employed for the catalytic activity tests and acquisition of kinetic data for NH_3 decomposition. For the lowest space velocity, 70 NmL min^{-1} g^{-1}, the higher helium concentration in the feed than for the rest of space velocities is due to the limitation of the mass flow controller to supply a lower flow rate.

Despite every exsolution characterization being studied for 700 ºC and 4 h, for *in-situ* catalytic tests, the exsolution time was increased to 5 h. This responds to two main reasons. First, the differences between the quartz horizontal reactor employed for *ex-situ* exsolutions and the NH_3 decomposition reactor set-up. Second, for NH_3 decomposition catalytic tests, the materials were previously sieved to obtain particle sizes between 200-400 µm, whereas the *ex-situ* exsolution treatments were performed with fine powders sieved under 200 µm. These two factors may condition the exsolution process.

Thus, to guarantee comparable size and populations of the exsolved NPs, the reduction time was increased. Focusing on 200-400 µm-sieved CGORu, similar (slightly lower) sizes and populations were achieved after 5 h exsolution treatment if compared to 4 h *ex-situ* reduction (see **Figure S1**). Hence, the increase in the *in-situ* reduction time ensures exsolution parameters similar to those observed in the *ex-situ* experiments. CGOimpRu was also *in-situ* reduced for activation of Ru, under the same conditions employed for exsolution treatments.

Gas products were online analyzed with an Agilent 7890 gas chromatograph equipped with two TCDs. The first TCD was used for He, H_2 and N_2 quantification after flowing through a Poraplot Q column (NH_3 backflush), followed by a Molsieve 13X column. For NH_3 analysis, the sample is injected by a split/splitless injector into a VolAmine phase column and quantified with the second TCD. These measures are performed every 4 min.

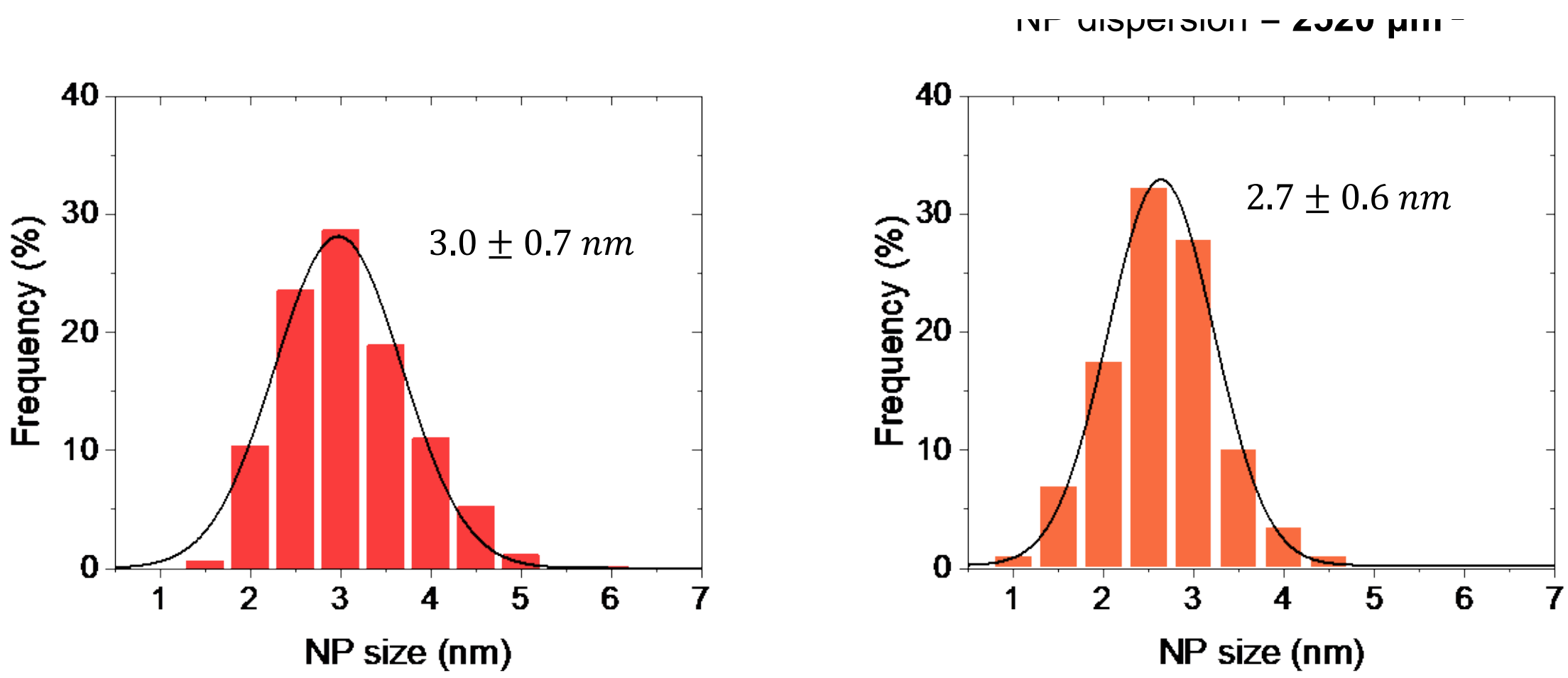

Figure S1. Comparison of exsolved NPs' mean size distribution and populations after *ex-situ* exsolution treatments at 700 ºC, 4 h (fine powders sieved under 200 µm) and *in-situ* exsolution treatments for catalytic tests at 700 ºC, 5 h (NP sizes between 200-400 µm).

NH_3 decomposition reaction is described by **Equation S2**[4]. Since no side reaction takes place, NH_3 conversion (X_{NH_3}) is calculated using the molar fractions of nitrogen (x_{N_2}) and NH_3 (x_{NH_3}) from chromatographic analyses, as described by **Equation S3**.

$$2NH_3 \rightarrow N_2 + 3H_2 \quad (\Delta H^0 = 92.4\ kJ\ mol^{-1}) \qquad \textbf{(Eq. S2)}$$

$$X_{NH_3} = \frac{2 \cdot x_{N_2}}{x_{NH_3} + 2 \cdot x_{N_2}} \qquad \textbf{(Eq. S3)}$$

Standard criteria for mass and heat transport limitations evaluation

For the determination of both heat and mass transport limitations and their possible impact on the reaction process, several standard criteria were employed[5], namely:

Weisz-Prater (intraparticle mass transport): $\frac{r^{eff} \cdot \rho_{cat} \cdot d_{cat}^2}{4 c_{NH3} \cdot D_{NH3}^{eff}} < 1$ **(Eq. S4)**

Anderson (intraparticle heat transport): $\frac{r^{eff} \cdot |\Delta H| \cdot \rho_{cat} \cdot d_{cat}^2}{4 \lambda^{eff} \cdot T} < \frac{0.75 \cdot R \cdot T}{E_a}$ **(Eq. S5)**

Mears (external mass transport): $\frac{r^{eff} \cdot \rho_{cat} \cdot d_{cat}}{h_m \cdot c_{NH3}} < 0.3$ **(Eq. S6)**

Mears (external heat transport): $\frac{r^{eff} \cdot |\Delta H| \cdot \rho_{cat} \cdot d_{cat}}{h_t \cdot T} < \frac{0.3 \cdot R \cdot T}{E_a}$ **(Eq. S7)**

Being $\boldsymbol{r^{eff}}$ the effective reaction rate; $\boldsymbol{\rho_{cat}}$ the catalyst density; $\boldsymbol{d_{cat}}$ the catalyst particle size; $\boldsymbol{c_{NH3}}$ the NH_3 concentration; $\boldsymbol{D_{NH3}^{eff}}$ the NH_3 effective diffusion; $\Delta \boldsymbol{H}$ the ammonia decomposition reaction enthalpy; $\boldsymbol{\lambda^{eff}}$ the effective thermal conductivity; $\boldsymbol{T}$ the temperature; $\boldsymbol{R}$ the ideal gas constant; $\boldsymbol{E_a}$ the activation energy; $\boldsymbol{h_m}$ the external mass transport coefficient; and $\boldsymbol{h_t}$ the external heat convection coefficient.

For these calculations, 400 ºC was considered at the lowest (25 mL min^{-1} NH_3+10 mL min^{-1} He) and highest (250 mL min^{-1} NH_3+50 mL min^{-1} He) reaction flows employed, to evaluate both extremes. The employed values -including those to calculate other parameters- and the criteria's results are depicted in the following **Table S2** and **Table S3**, respectively.

Table S2. Criteria results for internal and external transport, according to the values considered in **Table S3**.

**Description**	**Values**			
**Weisz-Prater criterion (Eq. S4)** (intraparticle mass transport)	$3.87\cdot10^{-10}$ **TRUE**	$3.87\cdot10^{-10}$ **TRUE**	$1.55\cdot10^{-9}$ **TRUE**	$1.55\cdot10^{-9}$ **TRUE**
**Anderson criterion (Eq. S5)** (intraparticle heat transport)	$1.71\cdot10^{-11}$ **TRUE**	$1.71\cdot10^{-11}$ **TRUE**	$3.16\cdot10^{-11}$ **TRUE**	$3.16\cdot10^{-11}$ **TRUE**
**Mears criterion (Eq. S6)** (external mass transport)	$1.36\cdot10^{-2}$ **TRUE**	$1.36\cdot10^{-4}$ **TRUE**	$5.43\cdot10^{-2}$ **TRUE**	$5.43\cdot10^{-4}$ **TRUE**
**Mears criterion (Eq. S7)** (external heat transport)	$2.74\cdot10^{-5}$ **TRUE**	$9.13\cdot10^{-6}$ **TRUE**	$1.26\cdot10^{-4}$ **TRUE**	$4.21\cdot10^{-5}$ **TRUE**

Being $\frac{0.75\cdot R\cdot T}{E_a} = 0.0328$ for the Anderson criterion comparative (**Equation S5**), and $\frac{0.3\cdot R\cdot T}{E_a} = 0.0131$ for the Mears criterion comparative (**Equation S7**). Therefore, all considered criteria reveal no mass or heat transport limitations in the performed tests.

Table S3. Parameter values considered for standard criteria calculations.

Parameter	Symbol	Units	Values			
**Feeding**						
**Flow**	F_0	mL min^{-1}	25	25	100	100
**Catalyst mass**	m_{cat}	g	0.5	0.5	0.5	0.5
**NH_3 feed concentration**	c_{0_NH3}	%	72	72	83	83
**Effective reaction rate**	r^{eff}	mol s^{-1} g^{-1}	$2.50\cdot10^{-5}$	$2.50\cdot10^{-5}$	$1.15\cdot10^{-4}$	$1.15\cdot10^{-4}$
**CGO-based catalysts properties**						
**Density**	ρ_{cat}	g cm^{-3}	7.09	7.09	7.09	7.09
**Effective thermal conductivity**	λ^{eff}	W cm^{-1} K^{-1}	0.02	0.02	0.05	0.05
**Particle size**	d_{cat}	nm	100	100	100	100
**Porosity**	por	%	40	40	40	40
**Tortuosity**	τ	-	1.58	1.58	1.58	1.58
**Process conditions**						
**Temperature**	T	K	673.15	673.15	673.15	673.15
**Pressure**	P	bar	1	1	1	1
**Gas properties**						
**NH_3 effective diffusion**	D_{NH3}^{eff}	cm^{-2}	0.877	0.877	0.877	0.877
**NH_3 concentration**	c_{NH3}	mol cm^{-3}	$1.30\cdot10^{-5}$	$1.30\cdot10^{-5}$	$1.50\cdot10^{-5}$	$1.50\cdot10^{-5}$
**Reaction enthalpy**	ΔH	J mol^{-1}	52112.6	52112.6	52112.6	52112.6
**Activation energy**	E_a	J mol^{-1}	128000	128000	128000	128000
**External mass transport coefficient**	h_m	m s^{-1}	$1.00\cdot10^{-4}$	$1.00\cdot10^{-2}$	$1.00\cdot10^{-4}$	$1.00\cdot10^{-2}$
**External heat convection coefficient**	h_t	W m^{-1} K^{-1}	50	150	50	150

Material	Ce (wt. %)	Gd (wt. %)	Ru (wt. %)	Rh (wt. %)
**CGO**	51.61	14.69	-	-
**CGORu**	64.10	14.92	3.36	-
**CGORuRh**	68.08	15.94	1.45	2.13
**CGORh**	54.82	13.16	-	3.84

Table S4. X-Ray Fluorescence results for CGO, CGORu, CGORuRh, and CGORh.

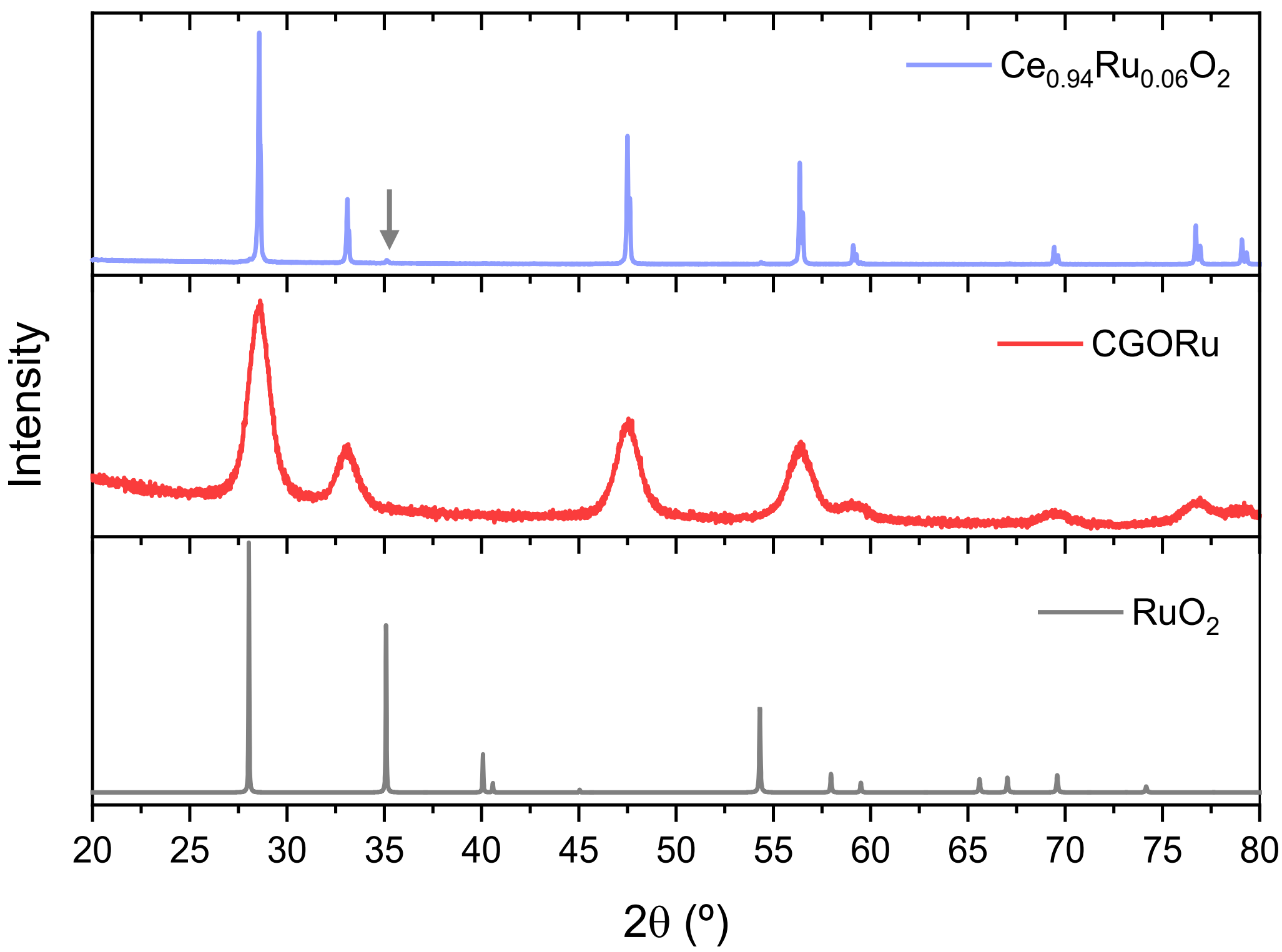

Figure S2. PXRD diffractogram comparative for synthesized $Ce_{0.94}Ru_{0.06}O_2$ (at 1100 ºC) and $(Ce_{0.8}Gd_{0.2})_{0.94}Ru_{0.06}O_2$ (CGORu) at 700 ºC. Without Gd in the structure, even at significantly higher sintering temperatures, minor amounts of RuO_2 can be appreciated (gray arrow), but not in Gd-doped material. This fact suggests that partial substitution of Ce with Gd eases the metallic introduction into the structure.

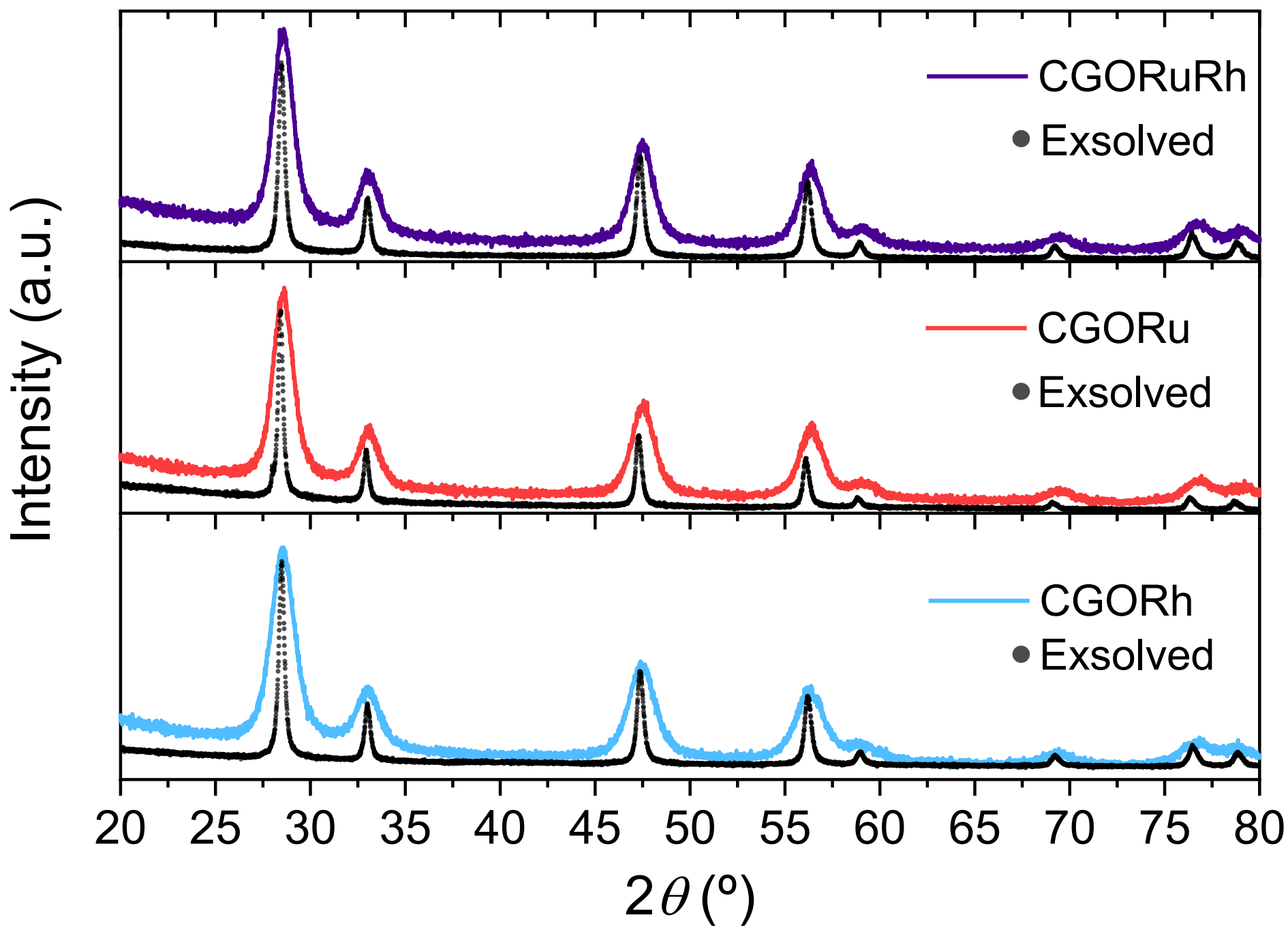

Figure S3. PXRD diffractograms of the pristine (700 ºC, 5 h in air) doped CGO materials (CGORuRh, CGORu and CGORh). No secondary phases (segregated metal oxides) were observed. Comparison with reduced materials for exsolution is included.

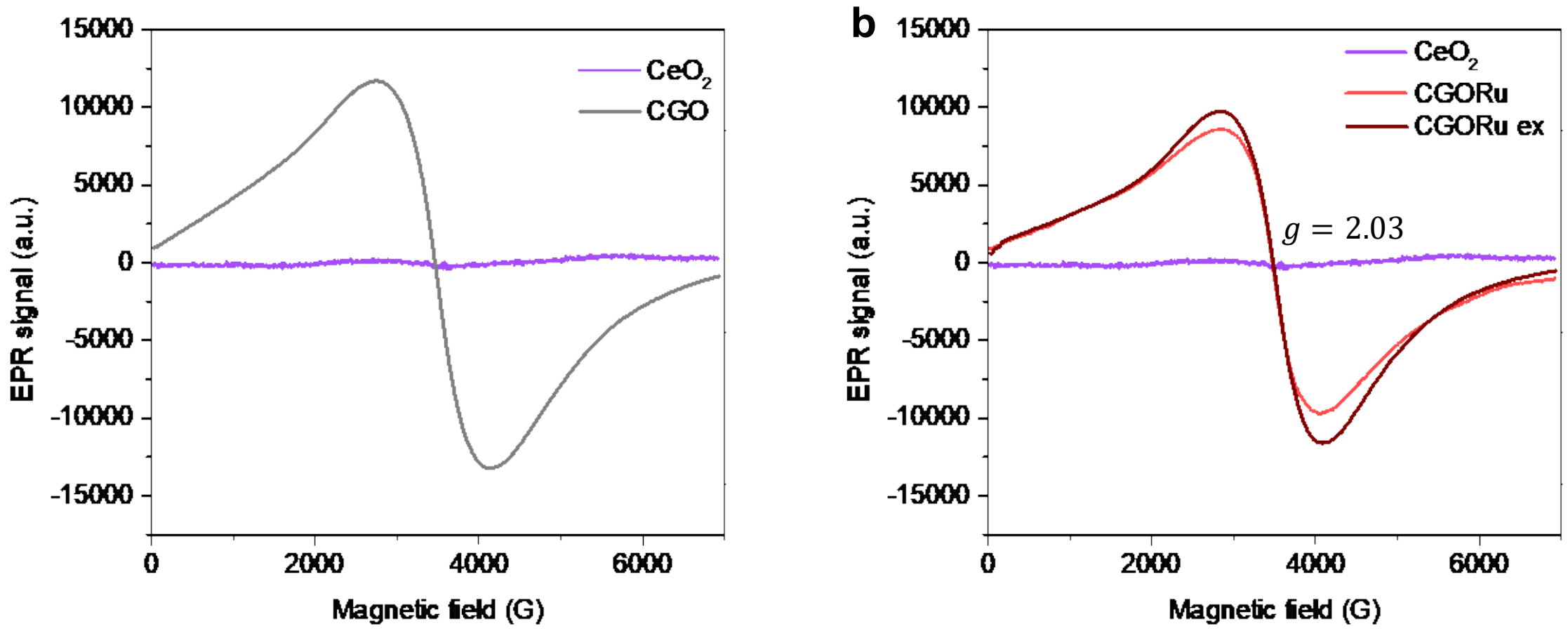

Figure S4. Mass-normalized EPR spectra for CeO_2 and CGO **(a)** and CGORu before and after exsolution, compared to CeO_2 **(b)**. The *g* factor was calculated at 9.85 GHz.

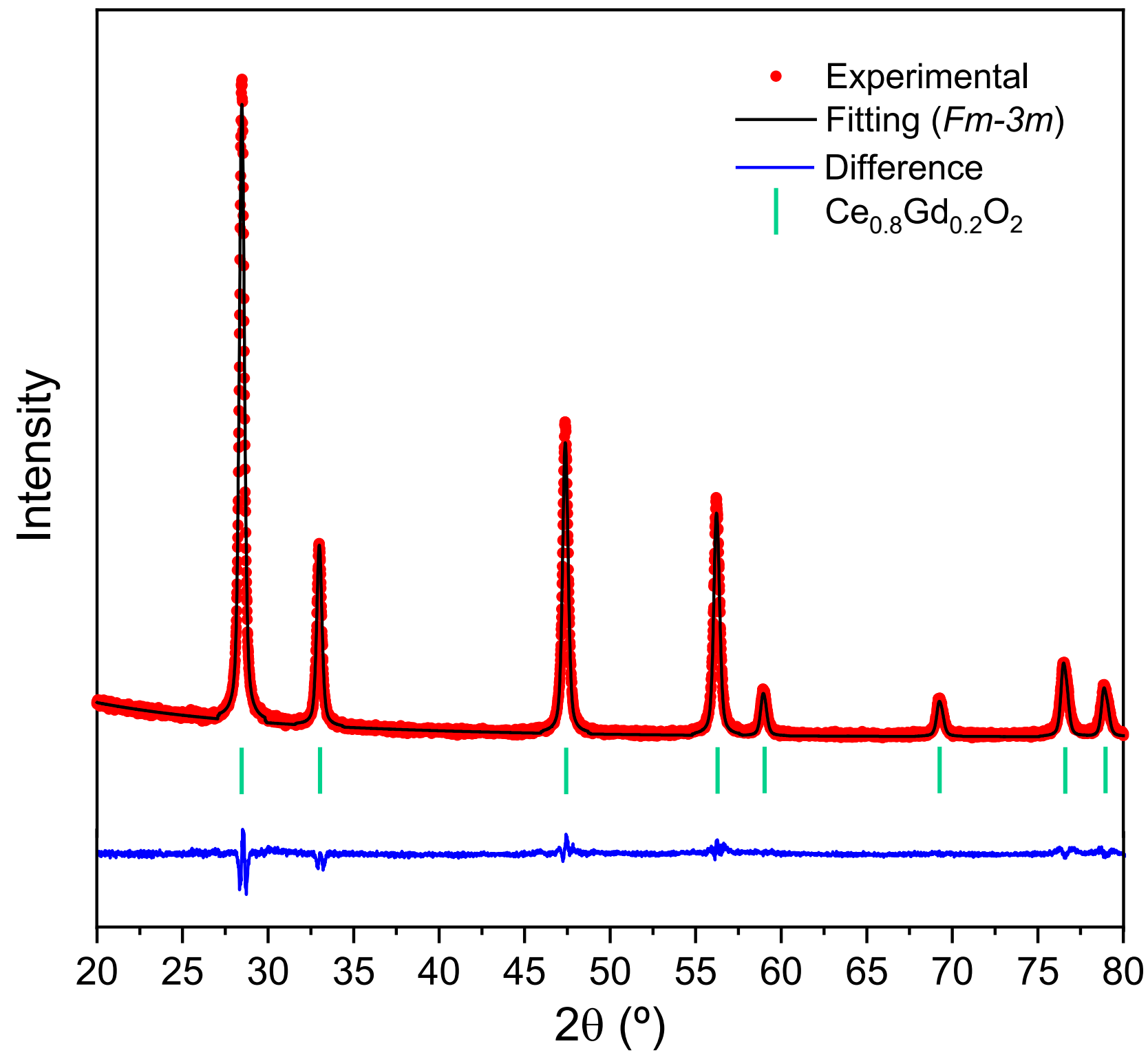

Figure S5. PXRD diffractogram and Rietveld refinement for as-synthesized CGO at 700 ºC for 5 h in air. This material exhibits a cell volume of 159.34 $Å^3$ (lattice parameter, a = 5.421 Å).

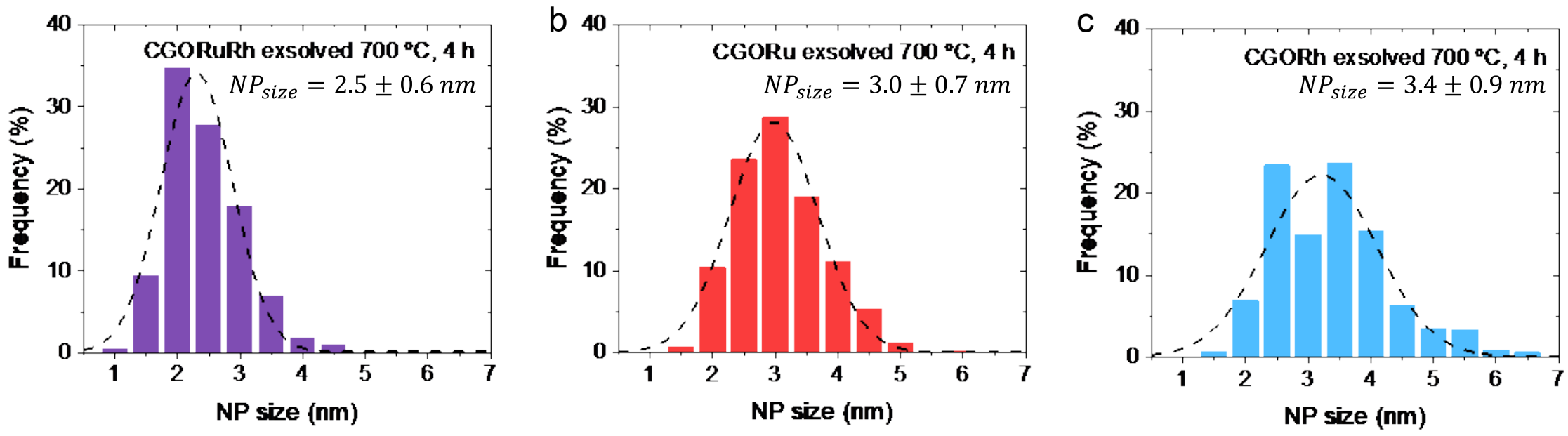

Figure S6. Size distribution histograms of the exsolved NPs at 700 ºC and 4 h, under 5% H_2/Ar flow for CGORuRh **(a)**, CGORu **(b)** and CGORh **(c)**.

Appendix S1

Calculations of exsolved metals (Ru, Rh, RuRh alloy), wt.%: $\boldsymbol{\xi_{ex}}$.

To obtain the exsolved atoms in the NPs (NP) from every reduced material (700 ºC, 4 h, 5% H_2/Ar), the calculations go as follows:

$$V_{NP} = \frac{4}{3}\pi\left(\frac{d}{2}\right)^3 \quad \textbf{(Eq. S8)}$$

$$NM_{atxNP} = \frac{V_{NP}\cdot\rho_{NM}}{A_{NM}\cdot(1.6605402\cdot10^{27})} \quad \textbf{(Eq. S9)}$$

Being $\boldsymbol{V_{NP}}$ the volume of the exsolved NP, obtained via the mean size (***d***) of the NPs (**Figure S6a-c**) for each material; $\boldsymbol{NM_{atxNP}}$ the number of noble metal atoms contained in an exsolved NP; $\boldsymbol{\rho_{NM}}$ noble metal atom density (12370 kg m^{-3} for Ru and 12400 kg m^{-3} for Rh); $\boldsymbol{A_{NM}}$ the atomic mass of the corresponding noble metal (101.07 u for Ru and 102.91 u for Rh). With the exsolved NPs populations for each material, ***NM atoms*** $\boldsymbol{\mu m^{-2}}$ can be calculated. Finally, with the BET area results, the mass of exsolved NM can be obtained and, thereby, the active superficial wt. % of NM in the catalysts ($\boldsymbol{\xi_{ex}}$). It is important to acknowledge that the calculated wt.% values are subject to uncertainties inherent in the image analysis process.

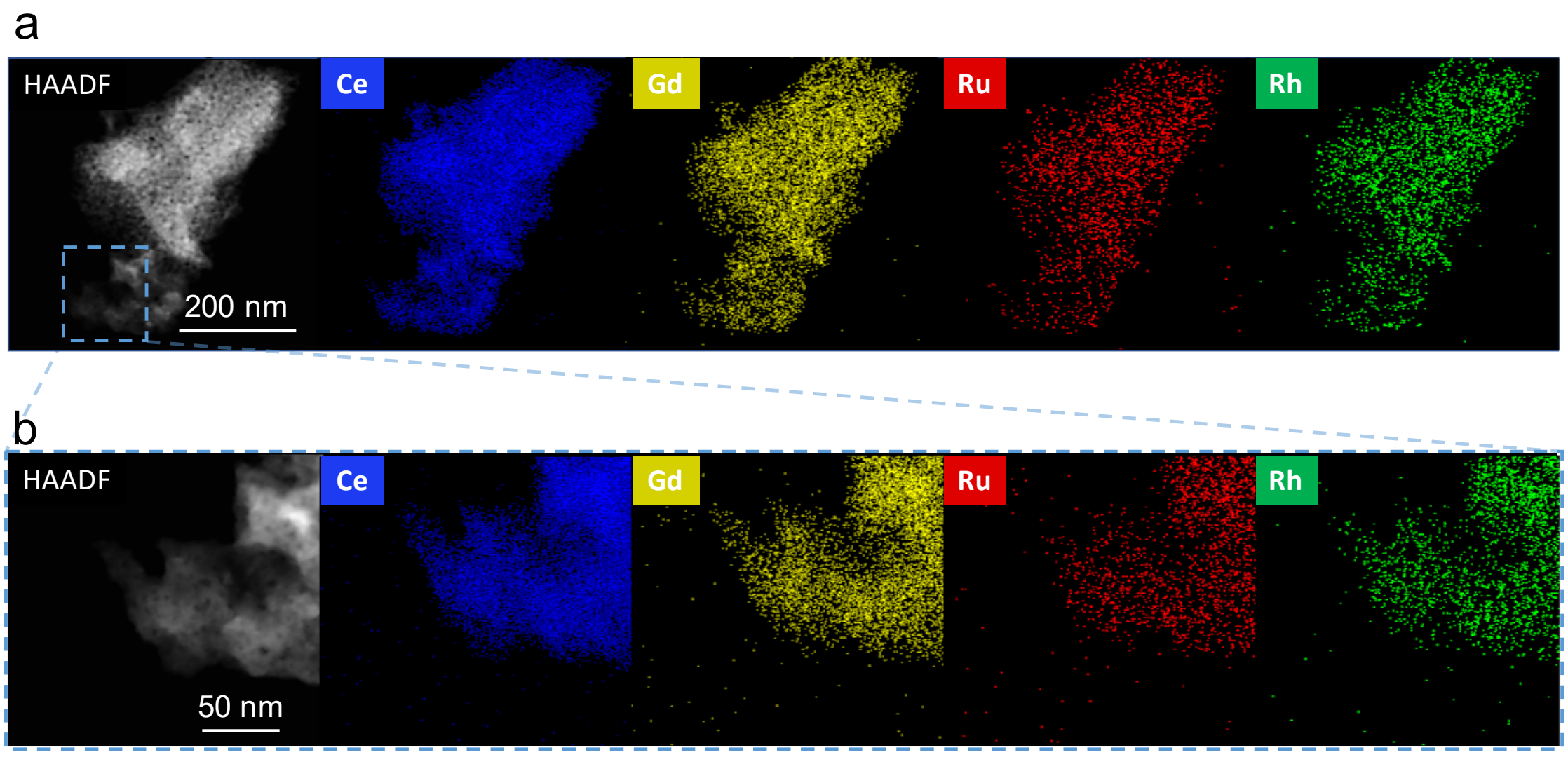

c

Element	Line Type	wt. %	$\sigma_{wt.\%}$
**Ce**	L-series	63.85	0.45
**Gd**	L-series	13.30	0.31
**Ru**	K-series	1.14	0.09
**Rh**	K-series	1.98	0.11
**O**	K-series	19.74	0.29

Figure S7. Pristine CGORuRh HAADF-STEM micrographs and XEDS map analyses, confirming the homogeneous distribution of Ru and Rh along the sample **(a)**. Higher detail of one of the analyzed regions **(b)**. Elemental analysis of pristine CGORuRh **(c)** from map micrographs in **(b)**.

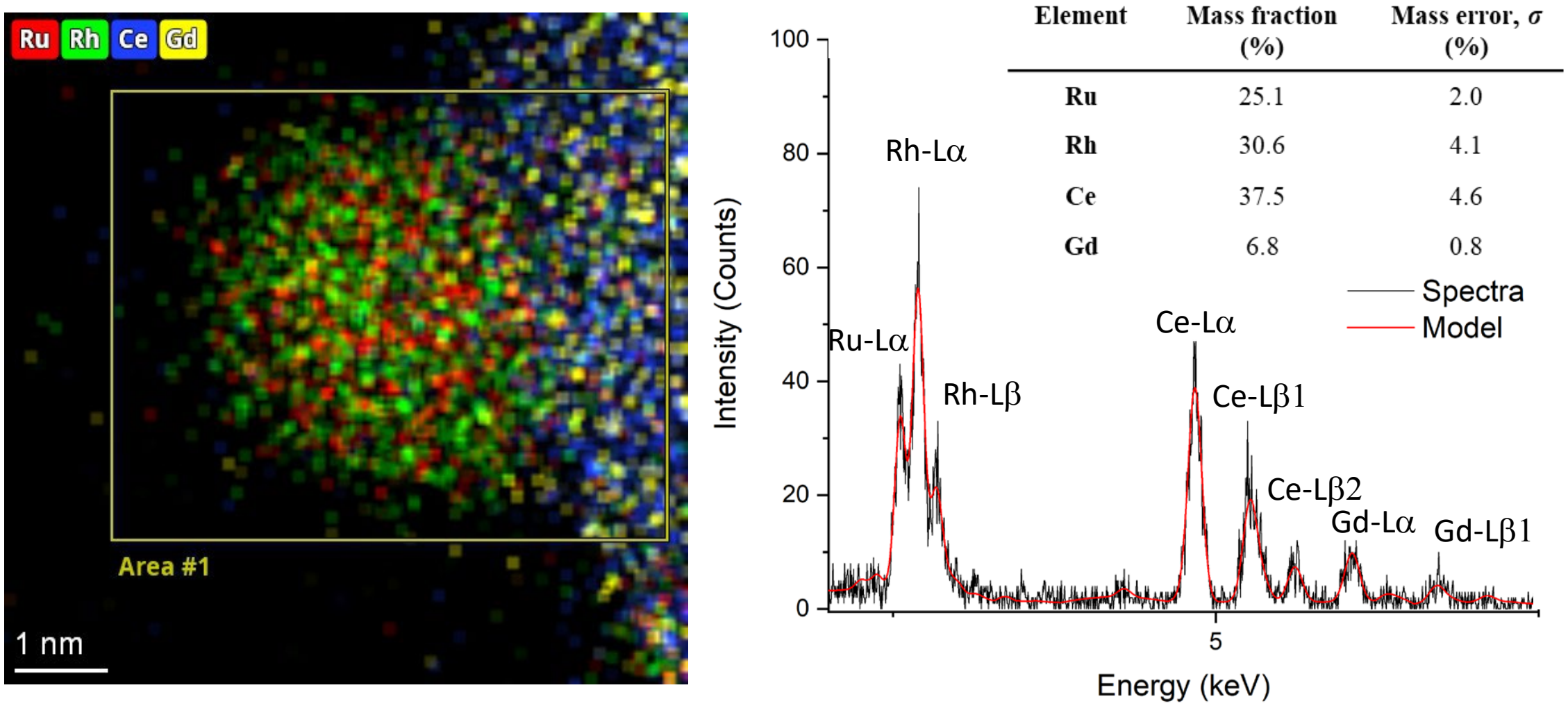

Figure S8. XEDS map image and elemental analysis of an exsolved NP from $(Ce_{0.8}Gd_{0.2})_{0.94}Ru_{0.03}Rh_{0.03}O_2$ (CGORuRh), after 700 ºC, 4 h reduction treatment under 5% H_2/Ar flow. Alloyed metallic Ru-Rh NPs are obtained, representing both metals similar mass percentages in the exsolved NP.

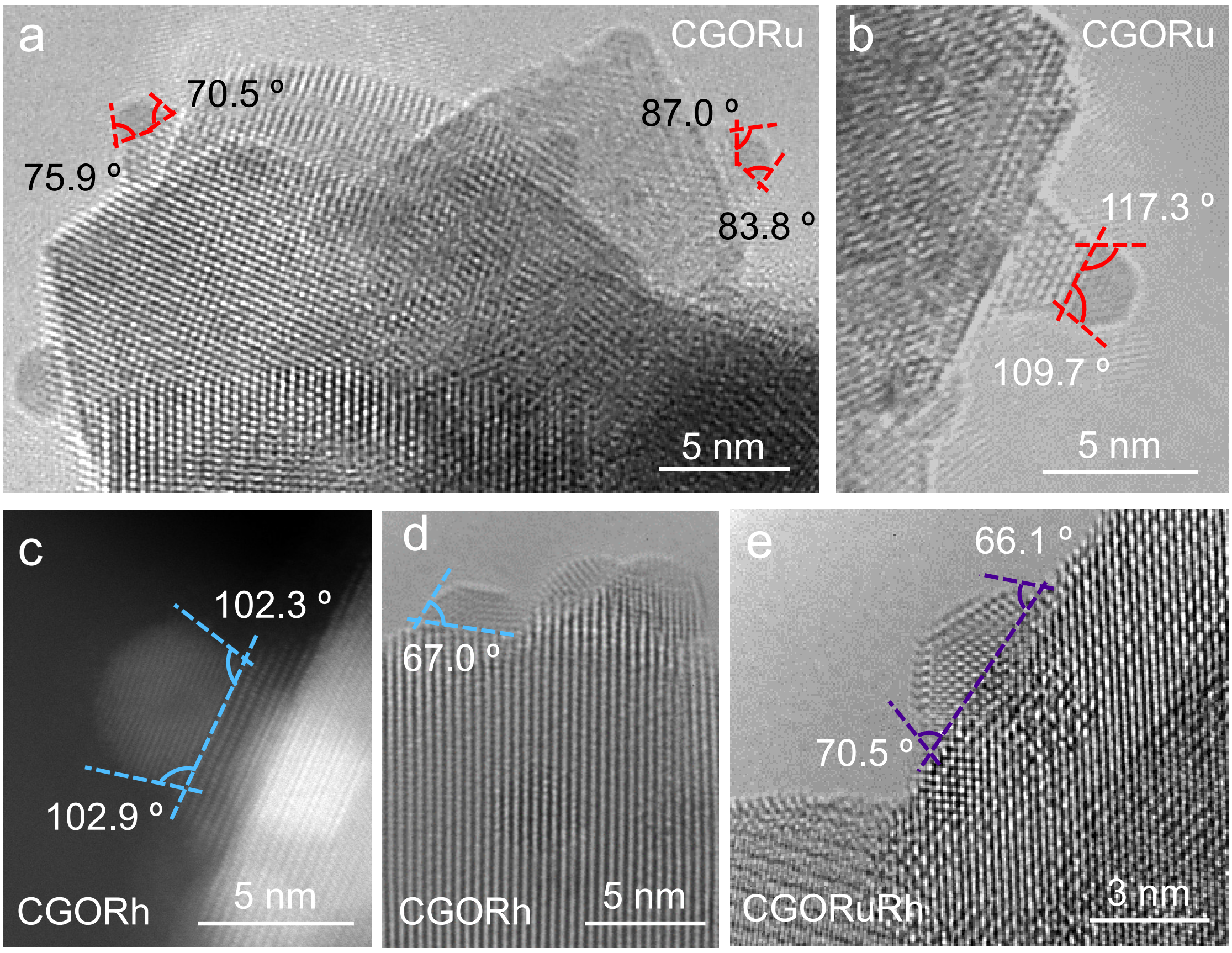

Figure S9. HRTEM micrographs and measured contact angles between exsolved NPs and support for: most commonly Ru exsolved NPs **(a)** and pedestal-emerged NPs **(b)** from CGORu; pedestal-emerged Rh NP **(c)** and most commonly observed NPs **(d)** from CGORh; exsolved RuRh NP from CGORuRh **(e)**.

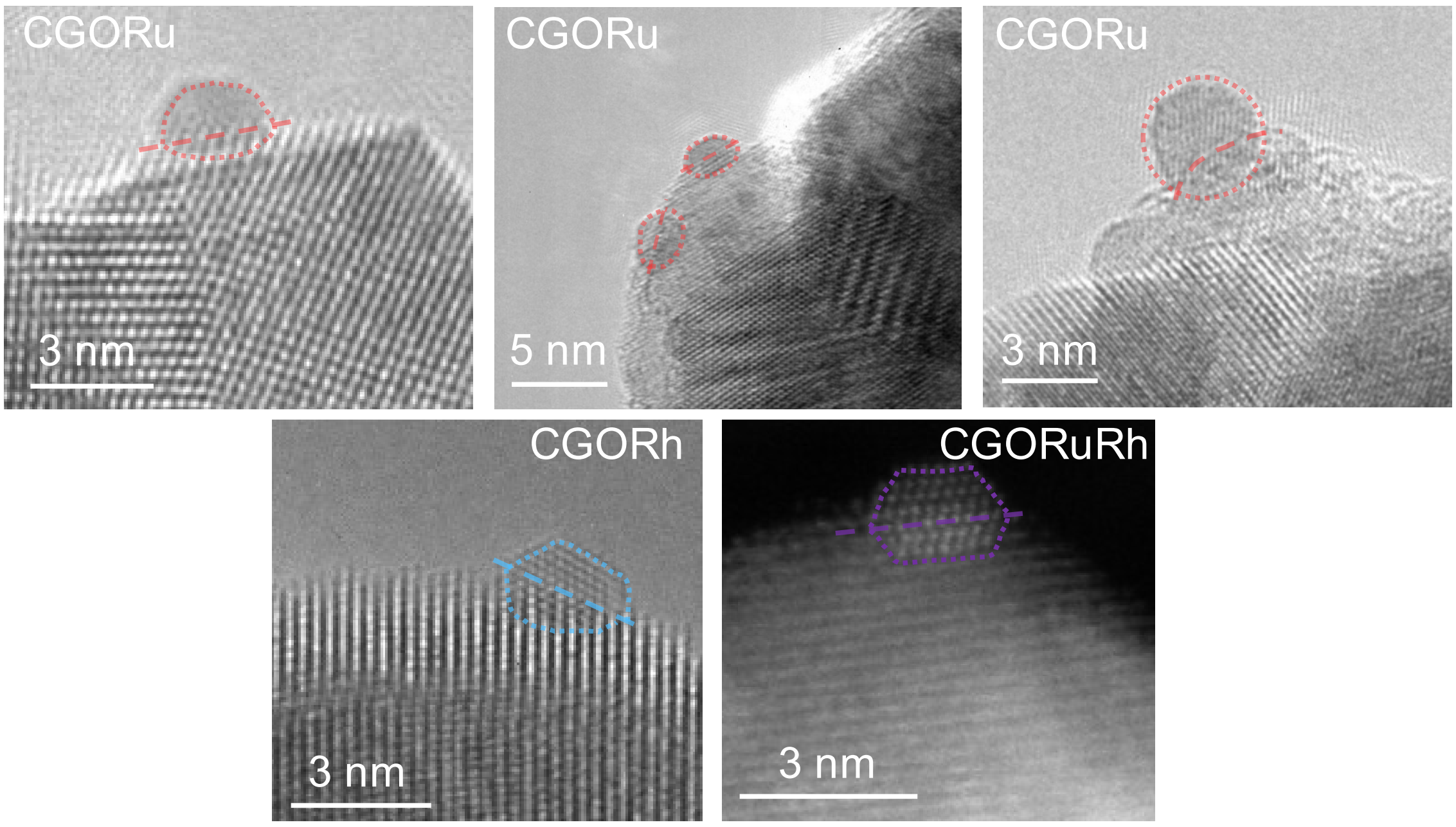

Figure S10. HRTEM micrographs depicting different examples of exsolved CGORu, CGORh and CGORuRh and indicating the anchored or embedded region of the NPs.

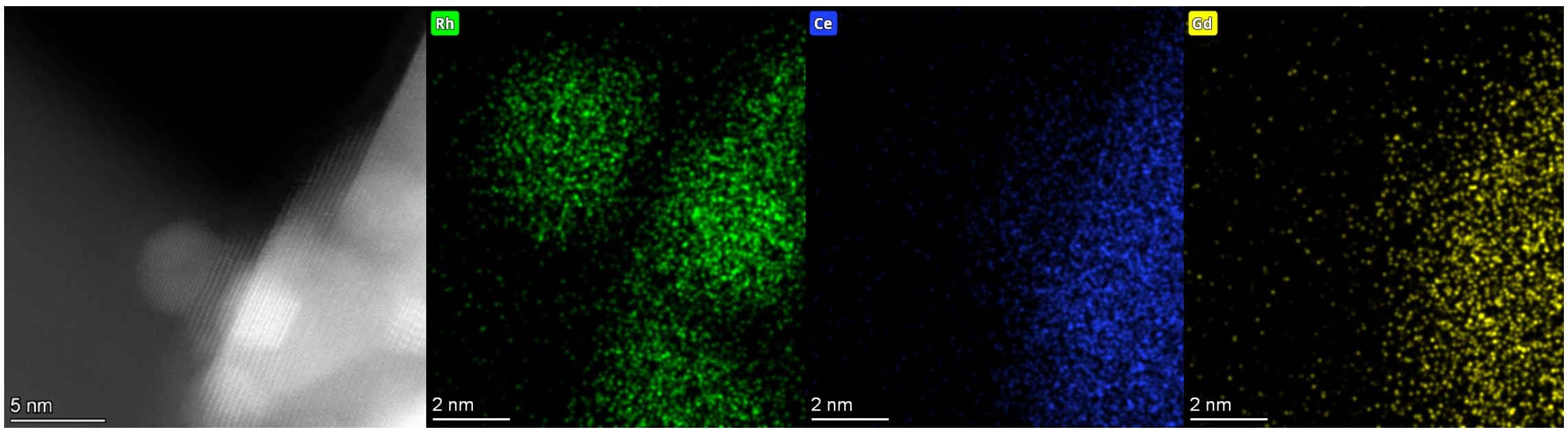

Figure S11. HAADF-STEM image and XEDS maps for exsolved NPs from $(Ce_{0.8}Gd_{0.2})_{0.94}Rh_{0.06}O_2$ (CGORh), after 700 ºC, 4 h reduction treatment under 5% H_2/Ar flow. Metallic Rh NPs are obtained.

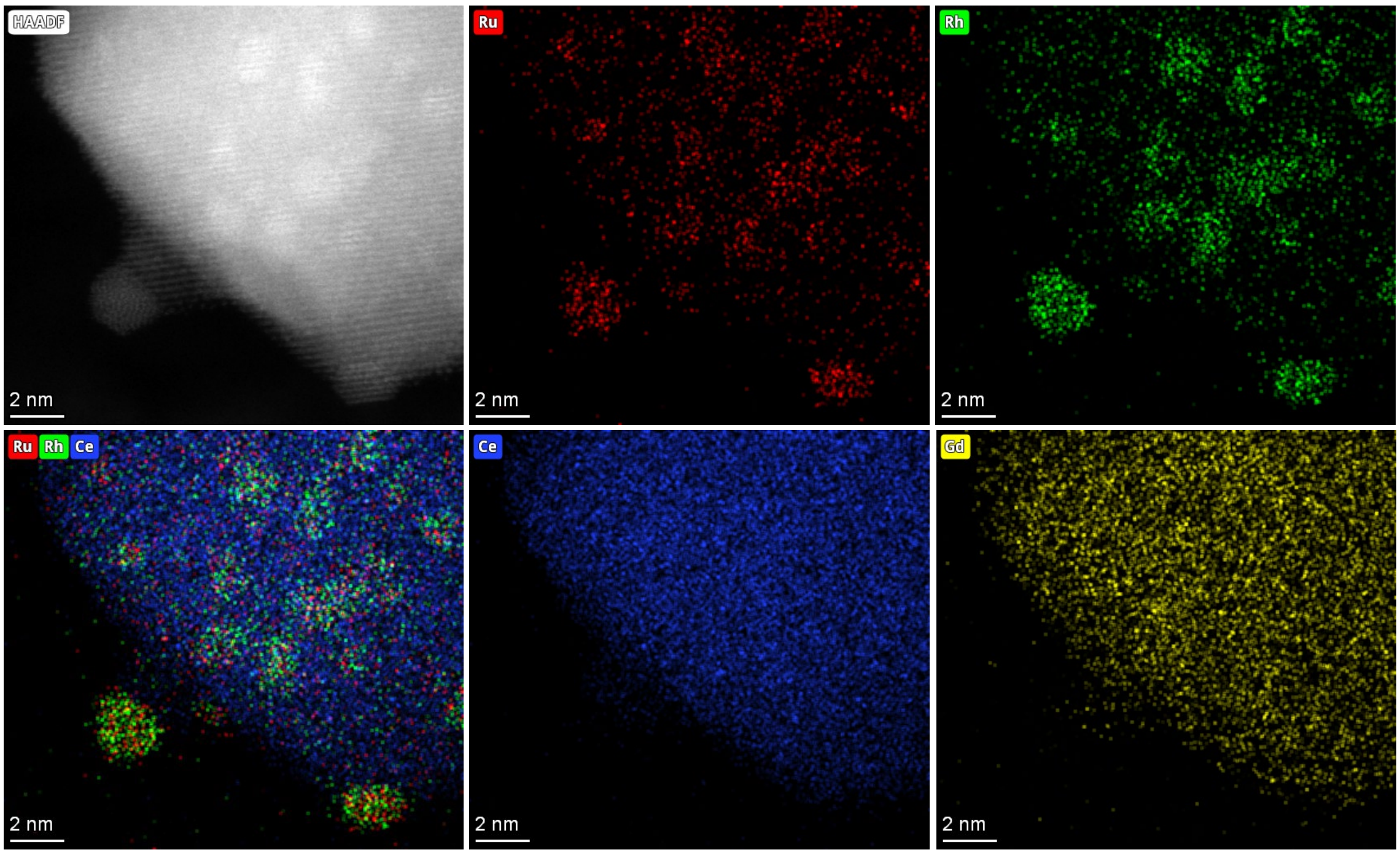

Figure S12. HAADF-STEM image and XEDS maps for exsolved NPs from $(Ce_{0.8}Gd_{0.2})_{0.94}Ru_{0.03}Rh_{0.03}O_2$ (CGORuRh), after 700 ºC, 4 h reduction treatment under 5% H_2/Ar flow. Alloyed metallic Ru-Rh NPs are obtained. Interestingly, as seen in CGORu, some NPs can be seen emerging from "pedestal-like" structures.

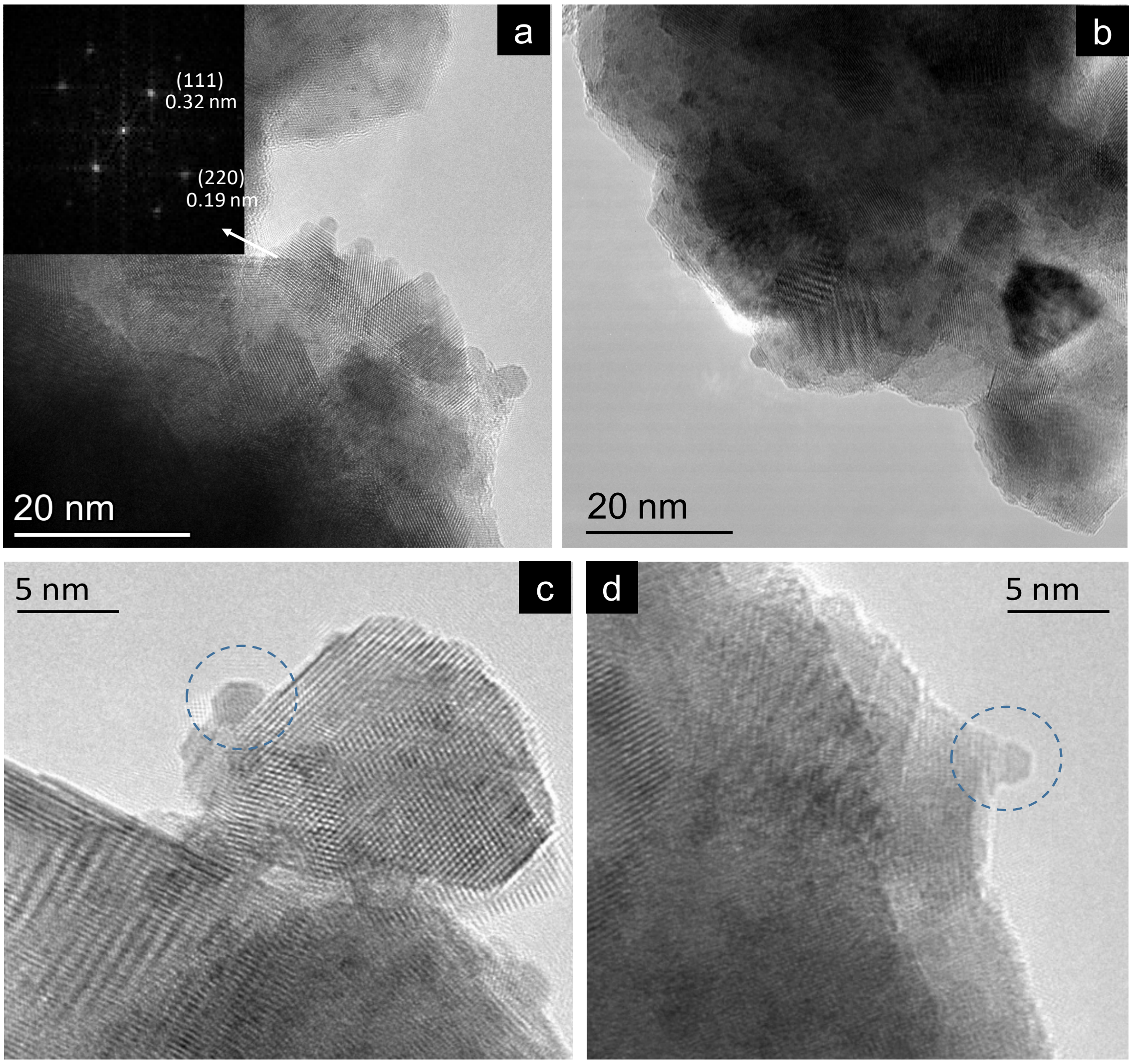

Figure S13. HRTEM micrographs of $(Ce_{0.8}Gd_{0.2})_{0.94}Ru_{0.06}O_2$ (CGORu), after 700 ºC, 4 h reduction treatment under 5% H_2/Ar flow. Exsolved metallic Ru NPs can be seen over the surface of the metal oxide **(a-b)**. Fast Fourier Transform (FFT) patterns of the metallic oxide show interplanar distances corresponding to (111) and (220) planes of $Ce_{0.8}Gd_{0.2}O_2$ (0.32 and 0.19 nm, respectively). HRTEM micrographs of exsolved CGORu, showing some faceted NPs **(c-d)**.

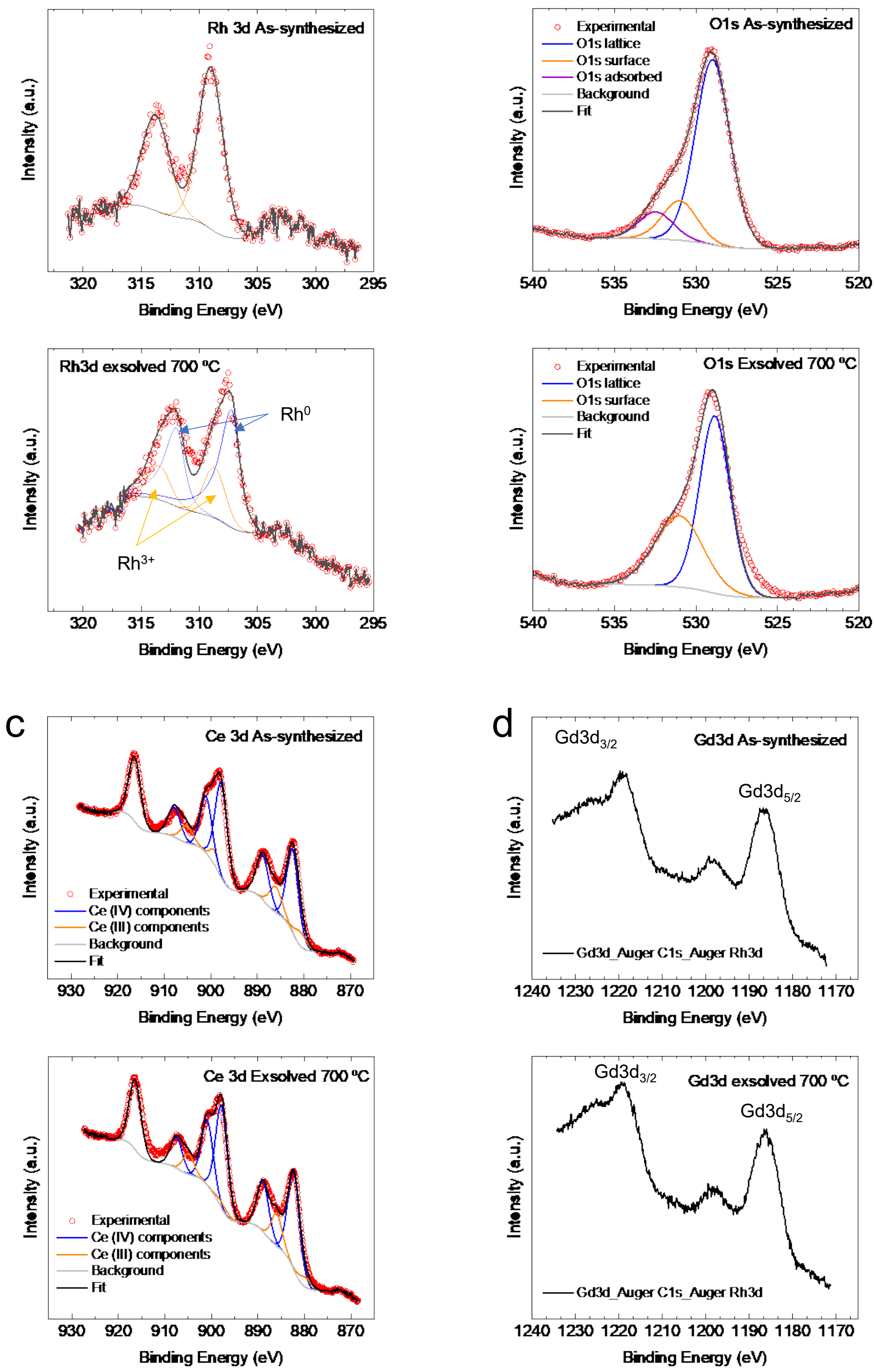

Figure S14. XPS spectra deconvolution of as-synthesized and exsolved CGORh material namely Rh 3d **(a)**, O 1s **(b)** and Ce 3d **(c)** regions. Gd 3d region **(d)** is also shown, and no changes are expected caused by thermal reduction for exsolution performed.

Sample	Composition	F_{2g} mode wavenumber (pristine, cm^{-1})	F_{2g} mode wavenumber (reduced, cm^{-1})
**CGORu**	$(Ce_{0.8}Gd_{0.2})_{0.94}Ru_{0.06}O_{2-\delta}$	459.8	463.2
**CGORuRh**	$(Ce_{0.8}Gd_{0.2})_{0.94}Ru_{0.03}Rh_{0.03}O_{2-\delta}$	460.9	462.1
**CGORh**	$(Ce_{0.8}Gd_{0.2})_{0.94}Rh_{0.06}O_{2-\delta}$	461.9	463.0
**CGO**	$Ce_{0.8}Gd_{0.2}O_2$	463.0	464.1

Table S5. Raman F_{2g} mode shift upon reduction (700 ºC, 4 h under 5% H_2/Ar flow) for every synthesized material.

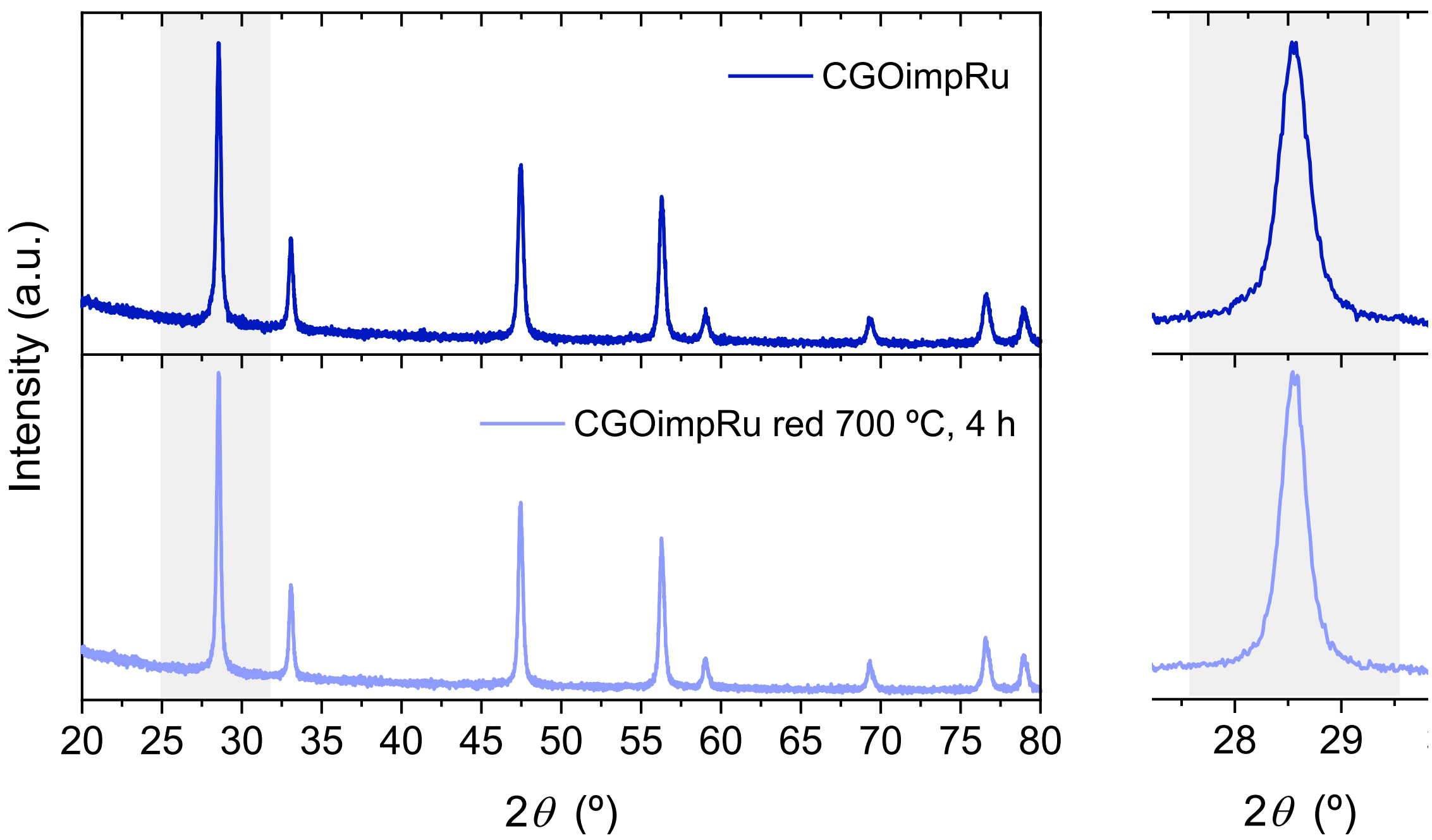

Figure S15. PXRD diffractograms of pristine and reduced (700 ºC, 4 h under 5% H_2/Ar flows) 1 wt.% CGOimpRu, including a zoom-up of the main signal, showing no significant lattice expansion after reduction.

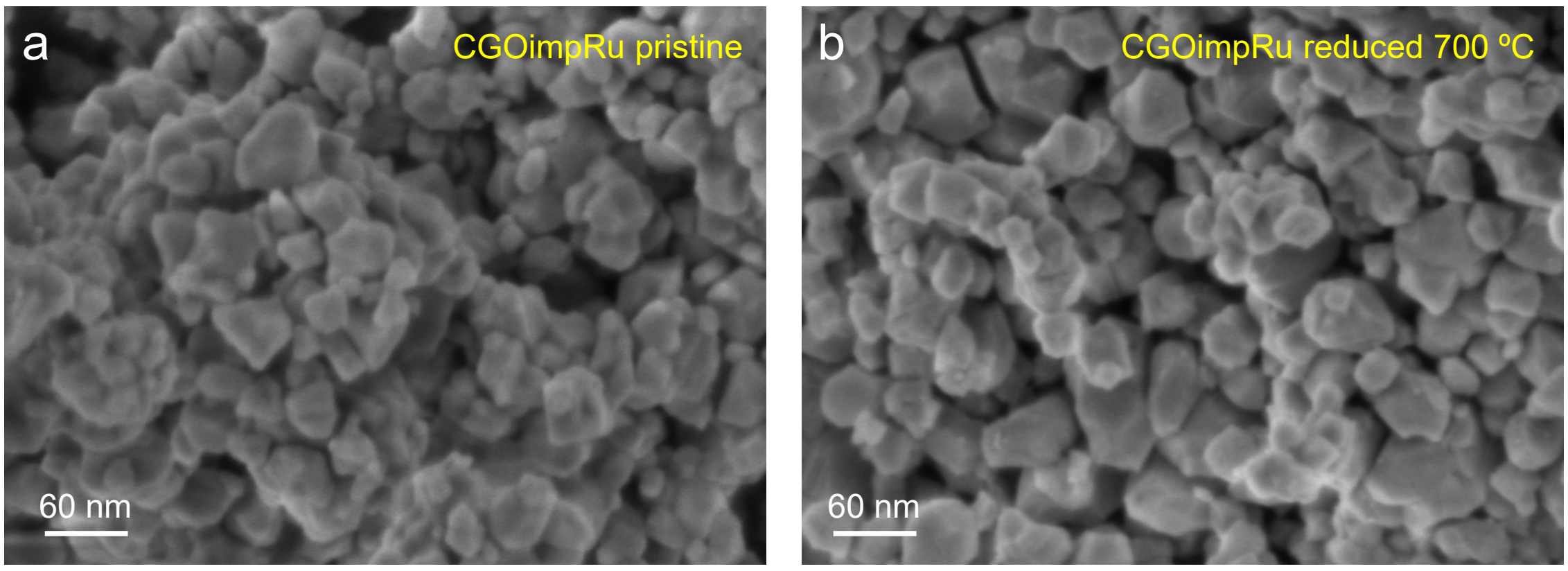

Figure S16. HRFESEM micrographs of CGOimpRu pristine **(a)** and reduced at 700 ºC, 4 h under 5% H_2/Ar **(b)**.

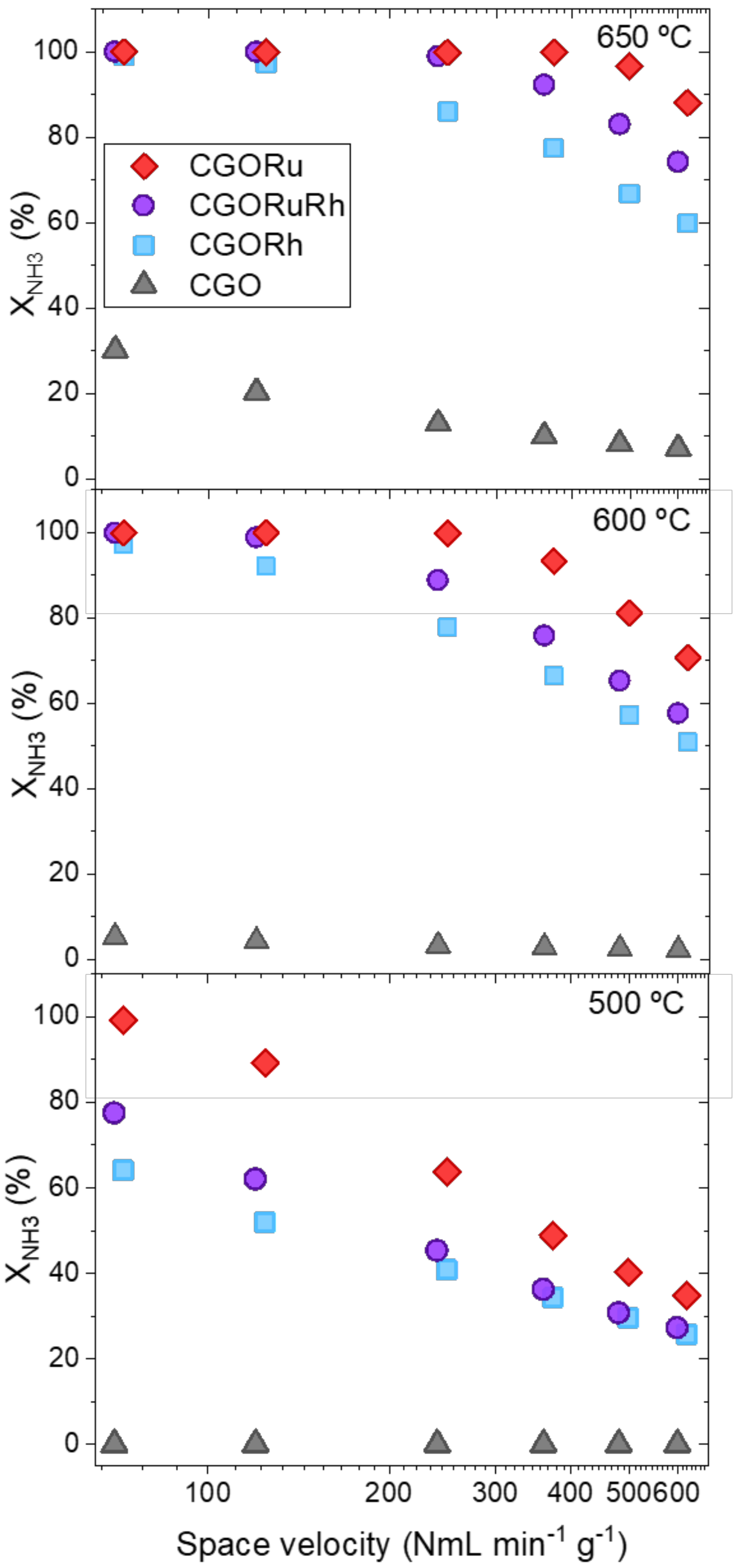

Figure S17. NH_3 decomposition conversion as a function of the space velocity at **(a)** 650, **(b)** 600, and **(c)** 500 ºC for undoped CGO, CGORu, CGORuRh, and CGORh exsolved catalysts.

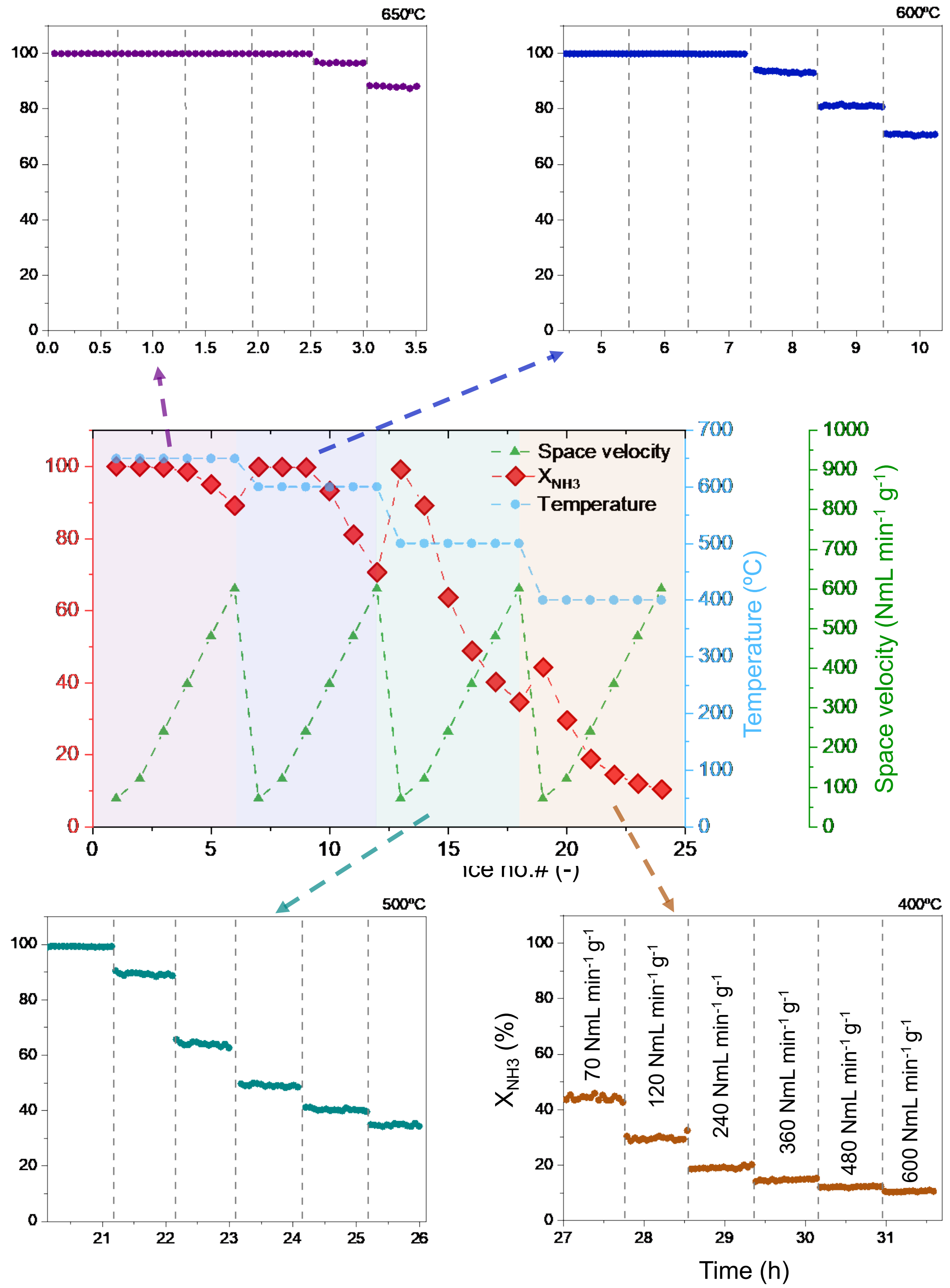

Figure S18. Representation of the stability of measured NH_3 conversions for CGORu at every temperature and space velocity tested. The central representation includes the measuring protocol followed. Each conversion value is calculated as an average value of the stable individual conversion values measured across each space velocity and temperature.

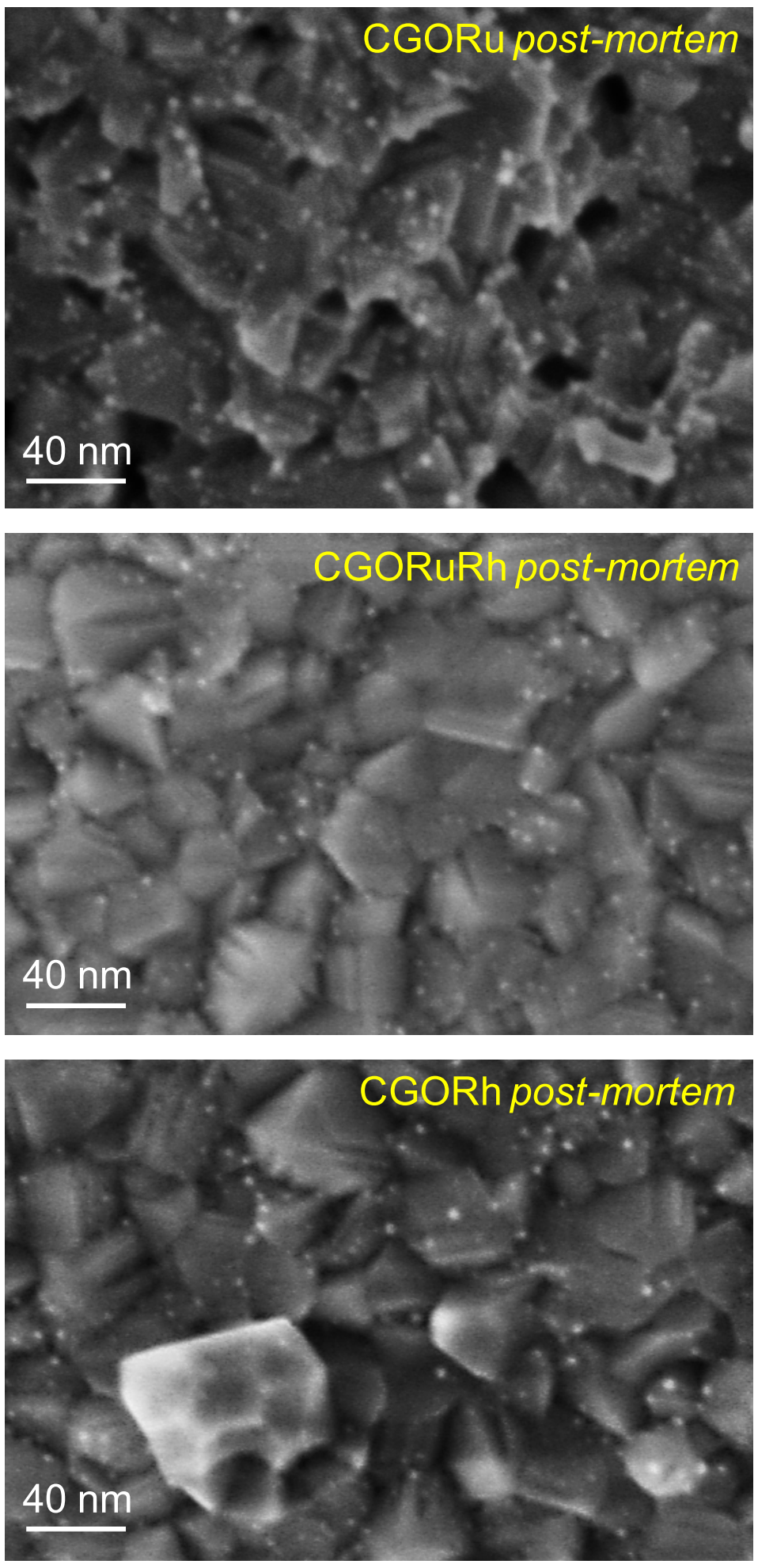

Figure S19. *Post-mortem* (NH_3 decomposition reactions) HRFESEM micrographs of the three tested catalysts.

Appendix S2

Turnover frequencies (TOF) calculations

TOF values for each exsolved catalyst at every employed temperature and space velocity were obtained through the following expression:

$$TOF = \frac{n_{NH3\_in} \cdot X_{NH3}}{n_{exposed\_metal} \cdot g_{cat} \cdot t}$$

being $\boldsymbol{n_{NH3\_in}}$ the introduced NH_3 moles at each space velocity (see **Methods**); $\boldsymbol{X_{NH3}}$ the NH_3 conversion at each condition; $\boldsymbol{n_{exposed\_metal}}$ the surface available metallic moles per g of catalyst; $\boldsymbol{g_{cat}}$ the employed catalyst grams (see **Methods**, 0.500 g each); and *t* the time.

For $\boldsymbol{n_{exposed\_metal}}$ calculations, previously obtained NP populations, mean sizes, and BET areas were needed. Considering semi-spheres without the base ($A = 2\pi r^2$) as the exposed fraction of the exsolved NPs (since the embedded fraction and the bulk of the NP are not surface-available, it is coherent to exclude them from the TOF calculations), the exposed metallic area of the exsolved NPs per catalyst gram ($\boldsymbol{m^2_{metal}/g_{cat}}$) was calculated. Consequently, considering spheric Ru and/or Rh atoms, the number of metallic atoms occupying the surface of the hemispheres (thus exposed metallic atoms), $\boldsymbol{n_{exposed\_metal}}$ was obtained. For RuRh alloys, metallic fractions from compositional analyses performed on the exsolved NPs (**Figure S8**) were considered.

	**CGORu**	**CGORuRh**	**CGORh**
**NP population (NPs µm^{-2})**	2986	3165	1686
**NP mean size (nm)**	3.0±0.7	2.5±0.6	3.4±0.9
**BET surface area (m^2 g^{-1})**	12.0	13.9	12.3
$\boldsymbol{m^2_{metal}/g_{cat}}$	0.52	0.40	0.37
$\boldsymbol{n_{exposed\_metal}}$	$6.5 \cdot 10^{-6}$	$5.2 \cdot 10^{-6}$	$4.9 \cdot 10^{-6}$
**TOF (s^{-1})**	**5.7**	**5.9**	**6.4**

Material	Weight %	X_{NH3} (%)	Space velocity (mL h^{-1} g^{-1})	TOF (s^{-1})	Ref. main manuscript
Ru-K/Al_2O_3	4.6	12	30,000	0.19	[91]
Ru-K/MgO	4.6	11	30,000	0.31	[91]
Ru/$Ca(NH_2)_2$	4.6	8	60,000	0.42	[93]
Ru/CNTs	4.7	17.7	30,000	0.60	[87]
Ru-K/$Mg_yAl_zO_n$	4.6	17	30,000	0.61	[91]
Ru/MgO com.	3.3	24.8	30,000	0.71	[89]
Ru/nano MgO	2.8	17.5	30,000	0.9	[88]
Ru-Cs/nano-MgO	2.8	23	30,000	1.18	[88]
Ru-$Ba(NH_2)_2$	4.4	20	60,000	1.29	[93]
Ru-Cs/MgO	2.0	15.2	60,000	2.9	[90]
Ru/MgO (111)	0.8	4.5	30,000	3.43	[89]
Ru-K/C	2.7	17.2	60,000	3.6	[90]
Ru-K/CNTs	4.8	63	60,000	4.8	[92]
**Exsolved CGORu**	**0.7**	**10.5**	**36,000**	**5.7**	**This work**
Ru/C12A7:e^-	2.2	70	15,000	6.9	[90]
Ru/C12A7:e^-	2.2	29.3	60,000	11.6	[90]

Table S6. Details of catalytic performances of various catalysts reported in other works (references referred to the main manuscript) for ammonia decomposition reaction at 400 ºC. Exsolved CGORu performance (this work) is included for comparative reasons.

Appendix S3

Simulated kinetic model

This work considered the following reaction rate equation based on the literature[6]:

$$NH_3 \rightarrow \frac{1}{2}N_2 + \frac{3}{2}H_2$$

Three different approaches were considered:

Power-law:

$$(-r_{NH3}) = k \cdot p_{NH3} \cdot p_{H2} \cdot \left(1 - \frac{1}{K_{eq}} \cdot \left(\frac{p_{N2} \cdot p_{H2}^3}{p_{NH3}^2}\right)\right)$$

RDS: N* desorption;

$$(-r_{NH3}) = \frac{k_B \cdot K_{NH3}^2 \cdot p_{NH3}^2}{\left(1 + K_{NH3} \cdot p_{NH3} + (K_{H2} \cdot p_{H2})^{\frac{3}{2}} \cdot (1 + \sqrt{K_{H2} \cdot p_{H2}})\right)^2}$$

RDS: first N-H cleavage;

$$(-r_{NH3}) = \frac{k_B \cdot K_{NH3} \cdot p_{NH3}}{\left(1 + K_{NH3} \cdot p_{NH3} + \sqrt{K_{H2} \cdot p_{H2}} + \sqrt{K_{N2} \cdot p_{N2}}\right)^2}$$

The model uses the Runge-Kutta methodology and plug-flow reactor to correct the composition, assuming a homogeneous distribution of the catalyst in the reactor:

$$\frac{dF_{NH3}}{dx} = -r \cdot \frac{m_{cat}}{L}$$

$$\frac{dF_{H2}}{dx} = 1.5 \cdot r \cdot \frac{m_{cat}}{L}$$

$$\frac{dF_{N2}}{dx} = 0.5 \cdot r \cdot \frac{m_{cat}}{L}$$

Knowing the outlet composition, the overall conversion was then compared with the experimental values:

$$error_i = |Xexp_i - Xmodel_i|$$

The sum of the deviations was minimized by varying the kinetic coefficients. The fitting procedure was simplified using the following equations:

$$k_B = k_{B,ref} \cdot \exp\left(-\frac{Ea_B}{R} \cdot \left(\frac{1}{T} - \frac{1}{T_{ref}}\right)\right)$$

$$K_i = K_{i,ref} \cdot \exp\left(-\frac{\Delta H_i}{R} \cdot \left(\frac{1}{T} - \frac{1}{T_{ref}}\right)\right)$$

Considering 500 °C as the reference temperature, the coefficient fitting was performed in two steps:

(1) the $k_{B,ref}$ and $K_{i,ref}$ were fitted using the results at 500 °C. Then, the fitting procedure considered four coefficients to minimize the error at this temperature.

(2) The results at the other temperatures (650, 600, and 400 ºC) were used to fit the activation energy (E_a) for each approach considered (**Tables S7-S9**).

This optimization methodology employs a Genetic Algorithm to minimize the objective error function under a set of defined constraints. The approach uses a highly customized configuration to enhance convergence and solution quality. Key parameters include a population size of 200 to ensure diversity, an elite count of 20 to preserve the best individuals, and a crossover fraction of 0.8 for strong recombination. Adaptive feasible mutation and tournament selection with a size of four are applied to balance exploration and exploitation. The algorithm runs for up to 200 generations, with a stall limit of 100 generations to prevent premature termination. Strict tolerances for function and constraint (10-*6*) improve precision, while parallel evaluation accelerates computation. Further, progress is monitored through plots of best fitness and population diversity. To enhance robustness, the optimization is repeated 20 times, recording the best solution across iterations.

Power-law

Parameter	CGORu	CGORh	CGORuRh	CGO
S_{BET} (m^2 g^{-1})	12.7	12.3	13.7	6.5
k_b, 500 ºC (mol g_{cat}^{-1} s^{-1})	$1.860 \cdot 10^{-4}$	$4.812 \cdot 10^{-5}$	$7.165 \cdot 10^{-5}$	$4.636 \cdot 10^{-8}$
E_a (kJ mol^{-1})	105.0	105.6	92.6	231.9

Table S7. Calculated kinetic parameters employing power-law approach for every exsolved catalyst studied.

RDS: N* desorption

Parameter	CGORu	CGORh	CGORuRh	CGO
S_{BET} (m^2 g^{-1})	12.7	13.7	12.3	6.5
k_b, 500 ºC (mol g_{cat}^{-1} s^{-1})	$2.941 \cdot 10^{-3}$	$6.183 \cdot 10^{-4}$	$7.248 \cdot 10^{-4}$	$6.118 \cdot 10^{-8}$
E_a (kJ mol^{-1})	39.6	37.8	43.2	215.7
$logK_{NH_3}$	-2.298			
ΔH_{NH3} (kJ mol^{-1})	77.8			
$logK_{H_2}$	-1.128			
ΔH_{H2} (kJ mol^{-1})	108.3			

Table S8. Calculated kinetic parameters employing RDS: N* desorption approach for every exsolved catalyst studied.

RDS: 1st N-H cleavage

**Parameter**	CGORu	CGORh	CGORuRh	CGO
S_{BET} ($m^2\ g^{-1}$)	12.7	13.7	12.3	6.5
k_b, **500 ºC** (mol $g_{cat}^{-1}\ s^{-1}$)	$7.574\cdot10^{-2}$	$1.626\cdot10^{-2}$	$1.972\cdot10^{-2}$	$4.658\cdot10^{-7}$
E_a (kJ mol^{-1})	50.7	51.2	63.0	224.0
$logK_{NH_3}$	-1.446			
ΔH_{NH3} (kJ mol^{-1})	40.8			
$logK_{H_2}$	0.778			
ΔH_{H2} (kJ mol^{-1})	128.7			
$logK_{N_2}$	-0.704			
ΔH_{N2} (kJ mol^{-1})	121.9			

Table S9. Calculated kinetic parameters employing RDS: 1st N-H cleavage approach for every exsolved catalyst studied.

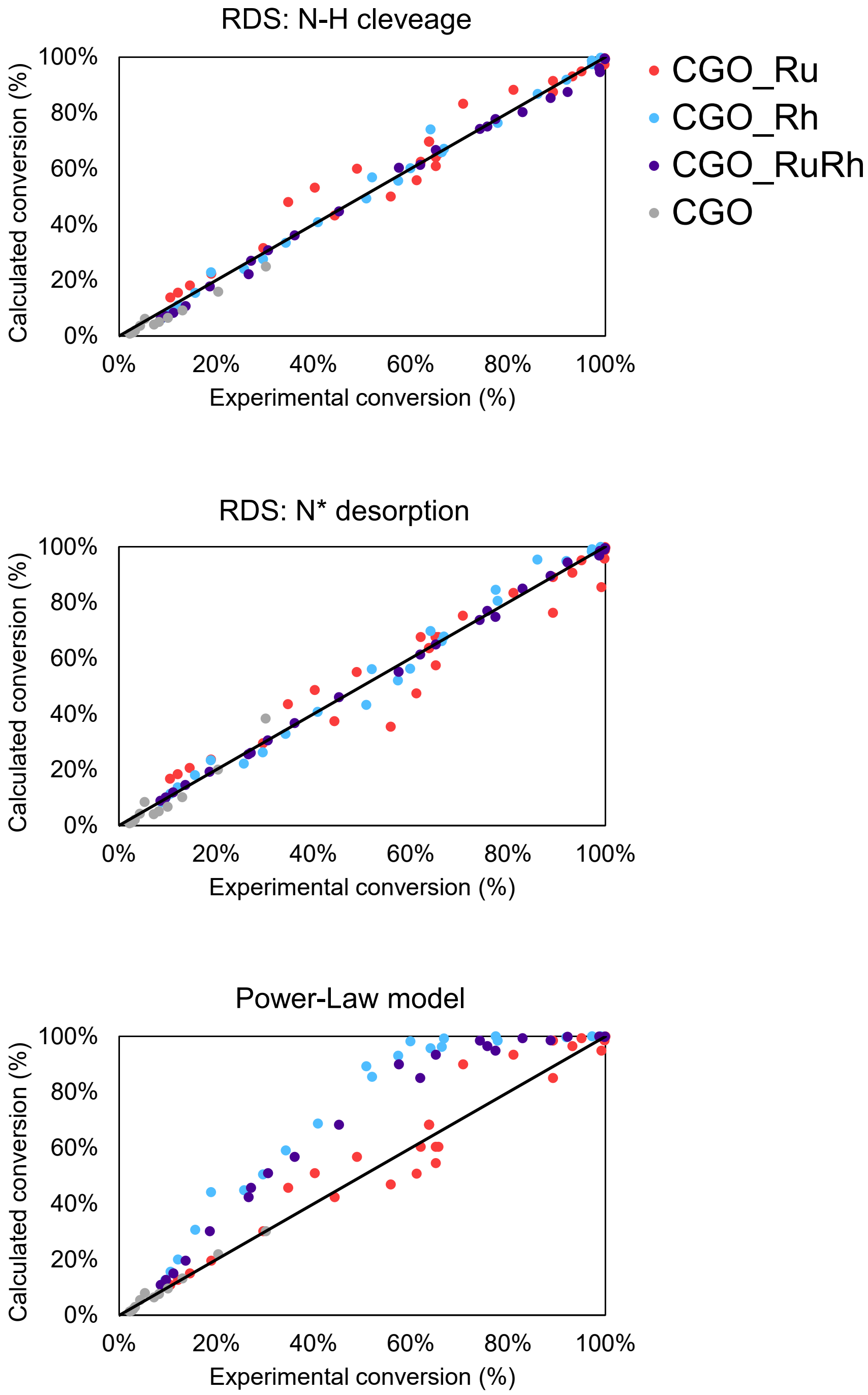

Figure S20. Fitting results obtained with the proposed simulated kinetic model, considering every different approach, for each exsolved catalyst and temperature. The best fitting results were obtained for RDS: 1st N-H cleavage approach (r = 99.4%), followed by RDS: N* desorption approach (r = 99.0%) and finally power-law (r = 94.3%).

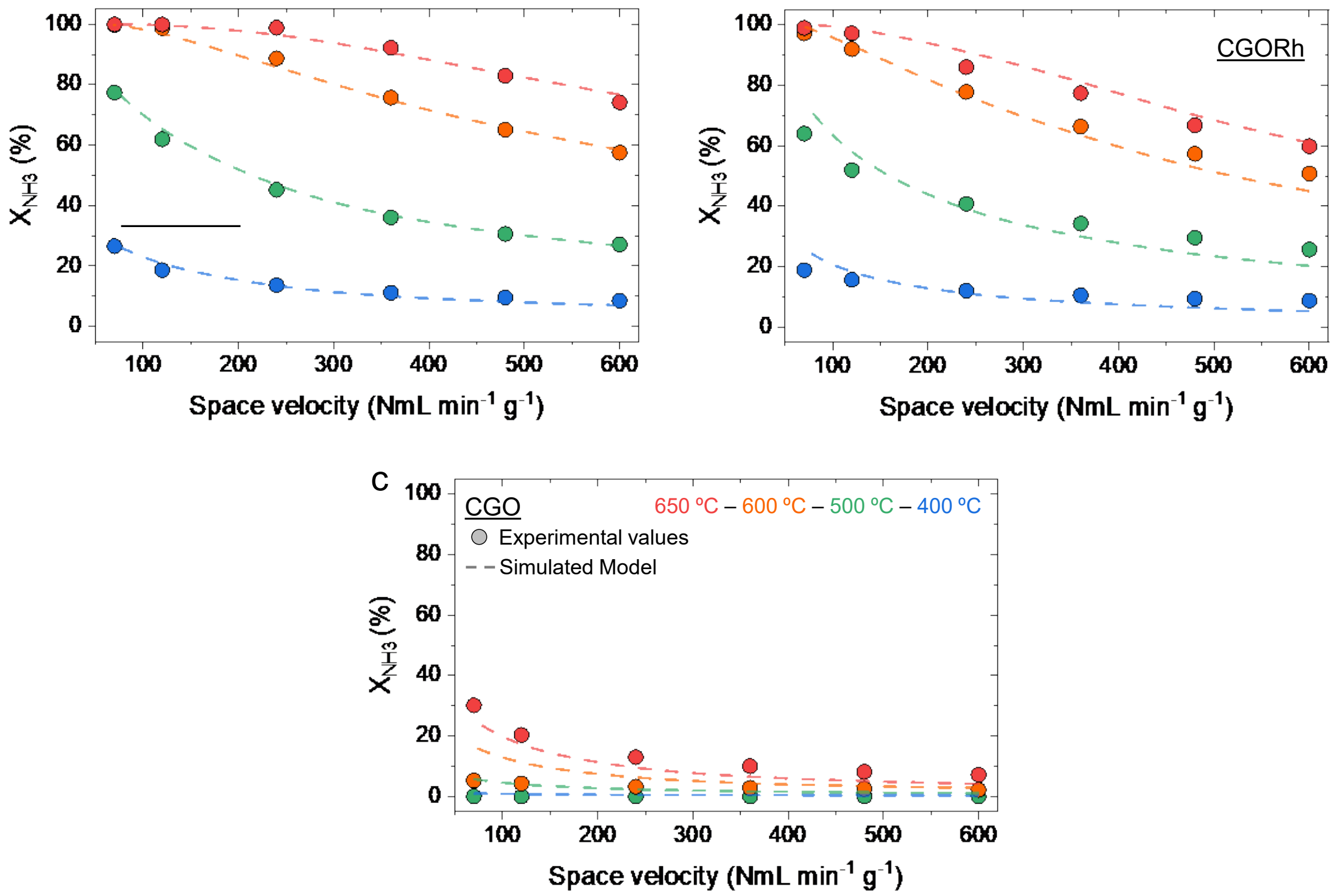

Figure S21. Experimental values (circles) and model values (lines) comparison for each exsolved catalyst considering RDS: 1st N-H cleavage approach. Each temperature is represented by a different color. The fittings show a notable accuracy with the experimental results.

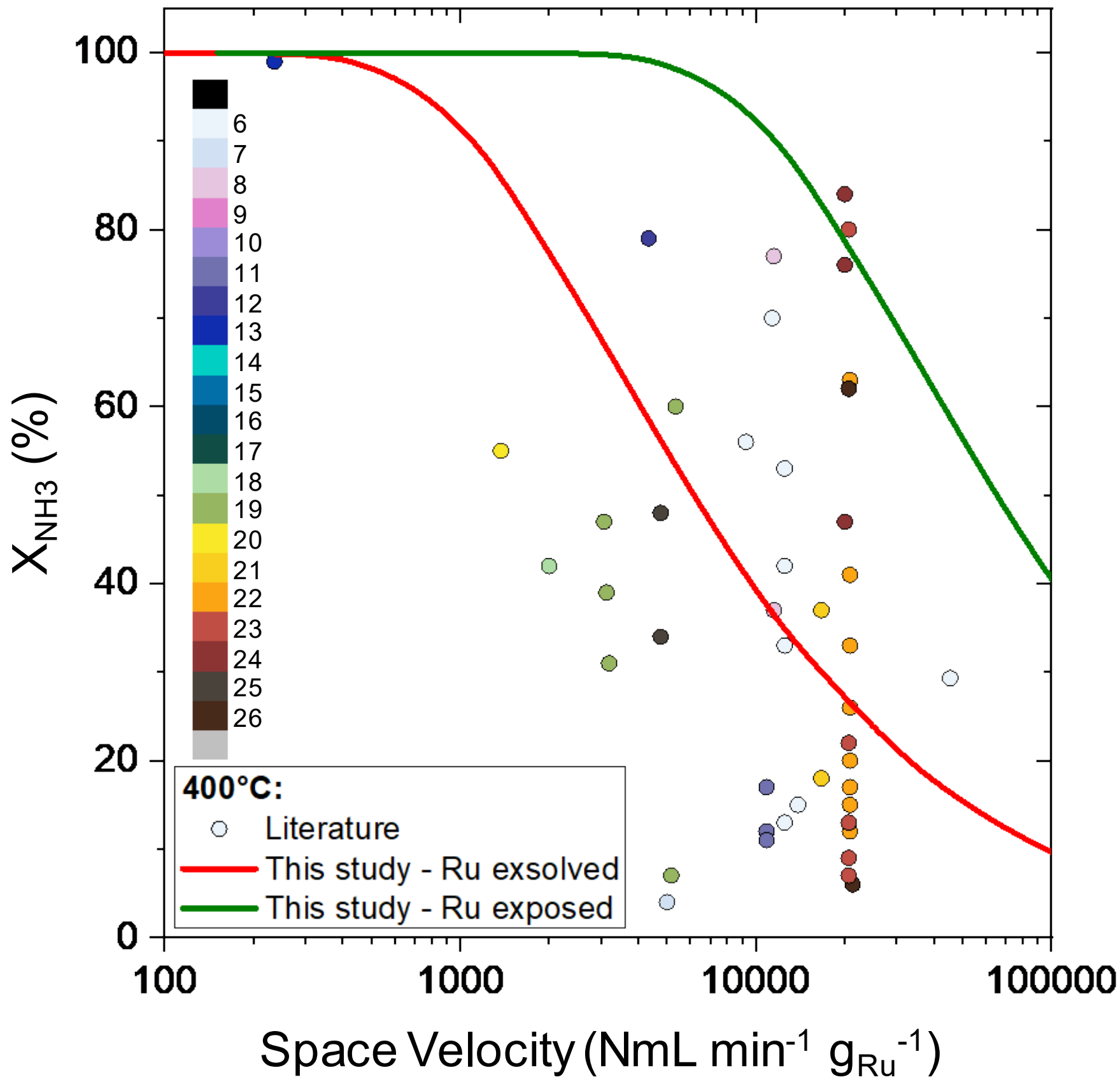

Figure S22. NH_3 conversion (X_{NH3}) studied as a function of Ru-based space velocity considering the Ru mass both exposed and exsolved, for CGORu at 400 ºC. These results are framed in a comparative with different literature results[6–26] (400 ºC). Exsolved Ru is calculated as explained in **Appendix S1** and exposed Ru estimation is included in **Appendix S2**.

Appendix S4

Evaluation of surface areas

Dense particle

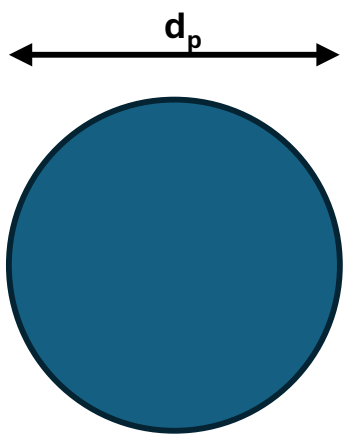

$$Area = 4\pi r^2 = \pi \cdot d_p^2$$

$$Volume = \frac{4}{3}\pi r^3 = \frac{\pi}{6} d_p^3$$

$$\frac{Area}{Volume} = \frac{6}{d_p}$$

Porous pellet particle

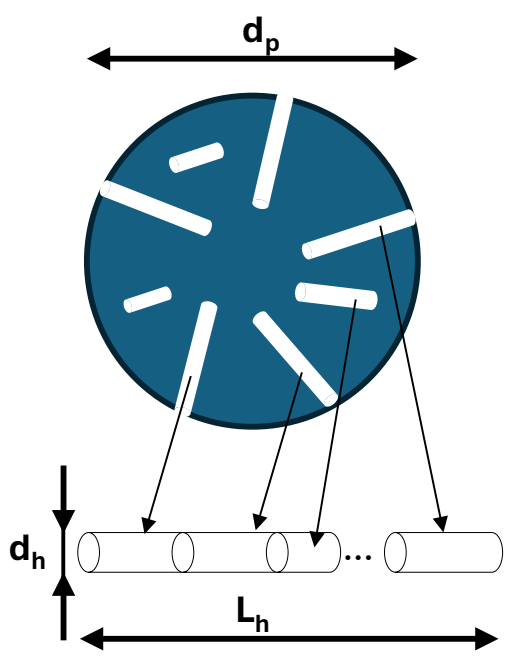

$$Area = Area_{ext} + Area_{int} = \pi \cdot d_p^2 + L_h \cdot \pi \cdot d_h$$

$$Volume = \frac{4}{3}\pi r^3 = \frac{\pi}{6} d_p^3$$

$$\frac{Area}{Volume} = \frac{6}{d_p} + \frac{6 \cdot L_h \cdot d_h}{d_p^3} = \frac{6}{d_p} + \frac{6 \cdot \frac{2}{3} \cdot \epsilon_{int} \cdot \frac{d_p^3}{d_h^2} \cdot d_h}{d_p^3} = \frac{6}{d_p} + 4 \cdot \epsilon_{int} \cdot \frac{1}{d_h}$$

$$\epsilon_{int} = \frac{Vol_h}{Vol_{tot}} = \frac{\frac{\pi}{4} d_h^2 \cdot L_h}{\frac{\pi}{6} \cdot d_p^3} \rightarrow L_h = \frac{2}{3} \cdot \epsilon_{int} \cdot \frac{d_p^3}{d_h^2}$$

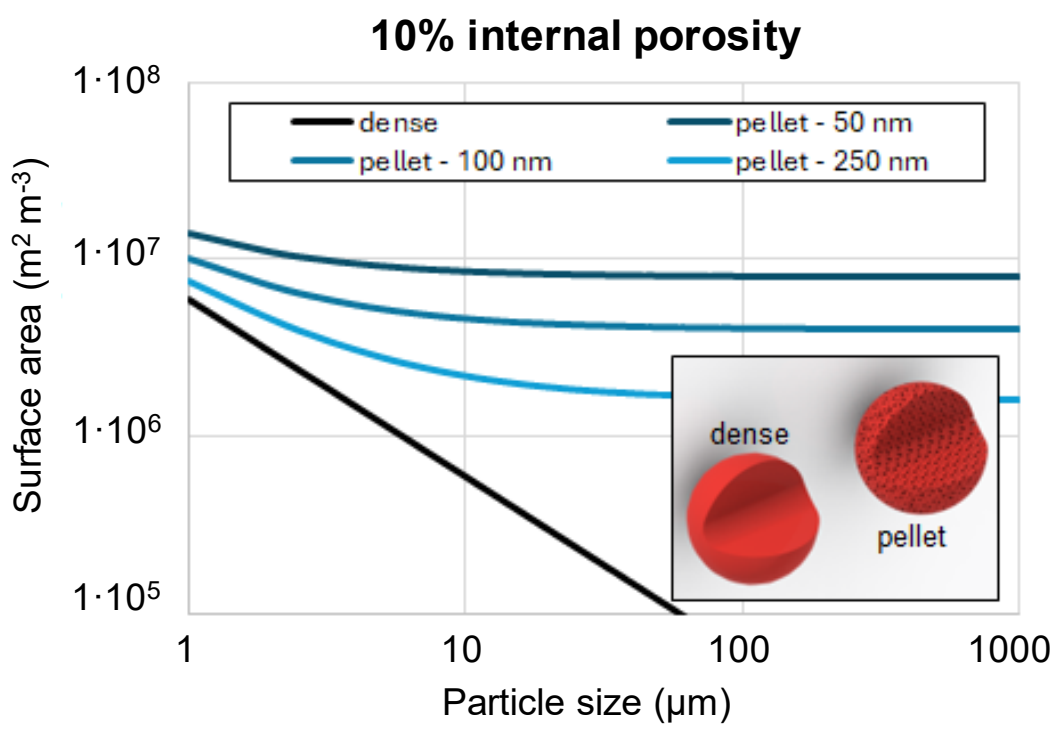

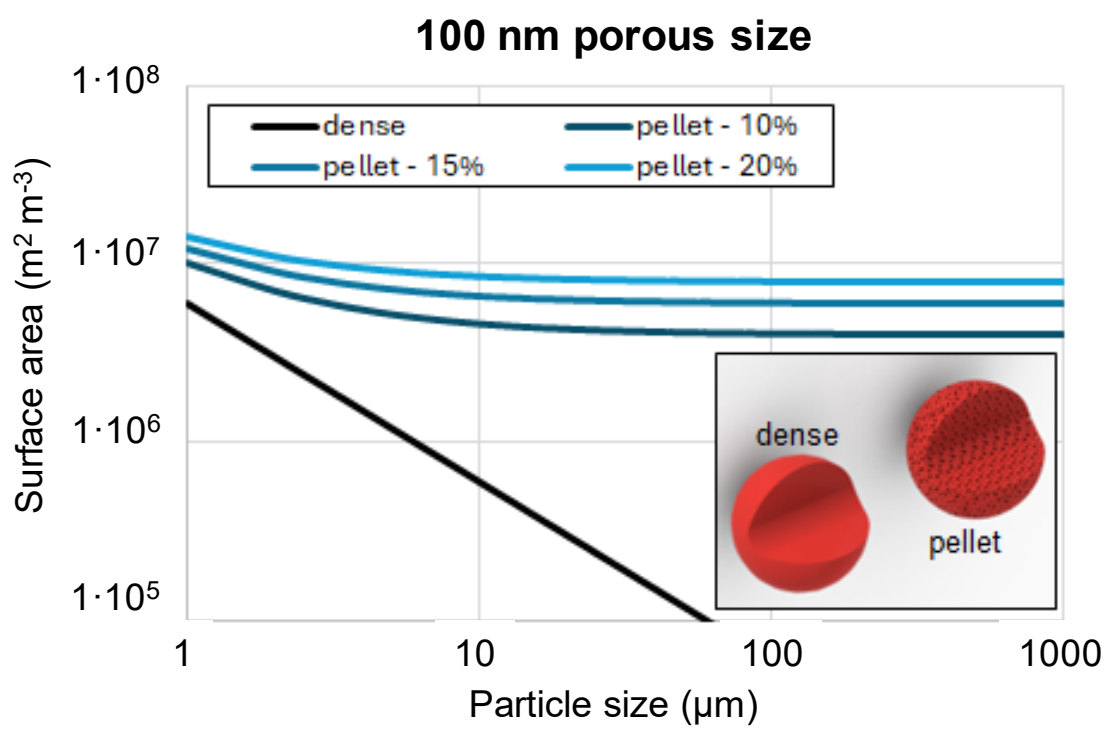

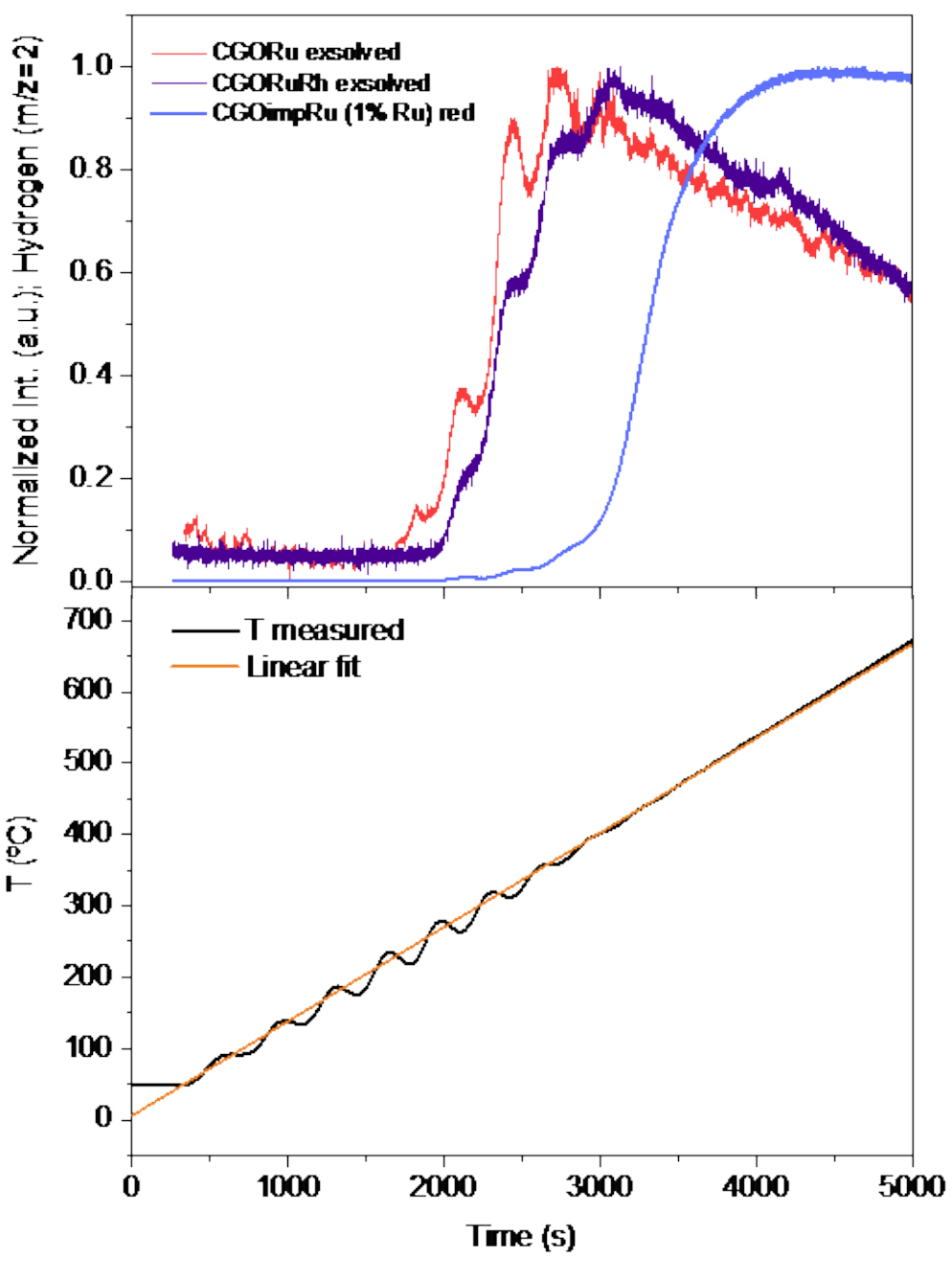

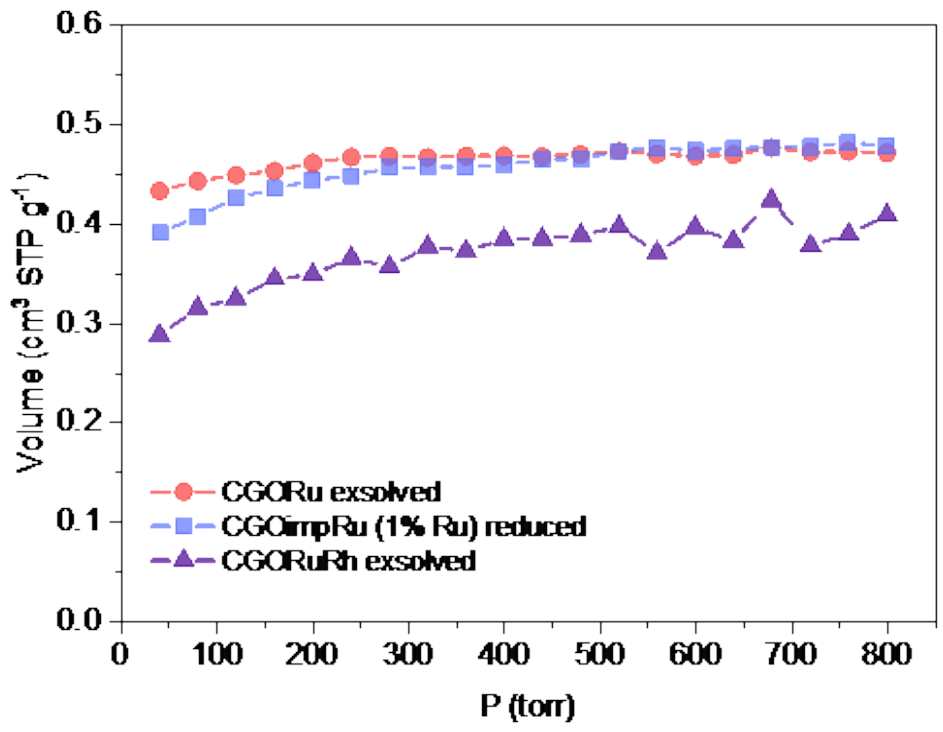

c

Material	Monolayer uptake	Active Area (/g metal)
**CGORu ex**	20.7 μmol/g	109.4 m²/g
**CGOimpRu red**	20.6 μmol/g	76.2 m²/g
**CGORuRh ex**	16.4 μmol/g	101.0 m²/g

Figure S23. NH_3-TPD analyses performed on exsolved CGORu, exsolved CGORuRh, and reduced CGOimpRu (1% Ru), obtained after NH_3 adsorption at 50 ºC for 1 h, followed by desorption under Ar flow using a heating rate of 9 ºC min^{-1} up to 700 ºC. Hydrogen evolution (m/z = 2) was continuously monitored by mass spectrometry. N_2 and NH_3 signals are not shown due to their low intensity and difficult interpretation. H_2 onsets: CGORu ≈ 260 ºC; CGORuRh ≈ 280 ºC; CGOimpRu ≈ 400 ºC **(a)**. CO chemisorption isotherms of exsolved CGORu, exsolved CGORuRh and reduced CGOimpRu **(b)**. Summary of the monolayer CO uptake and active metal surface area per gram of metal determined from the chemisorption measurements **(c)**.

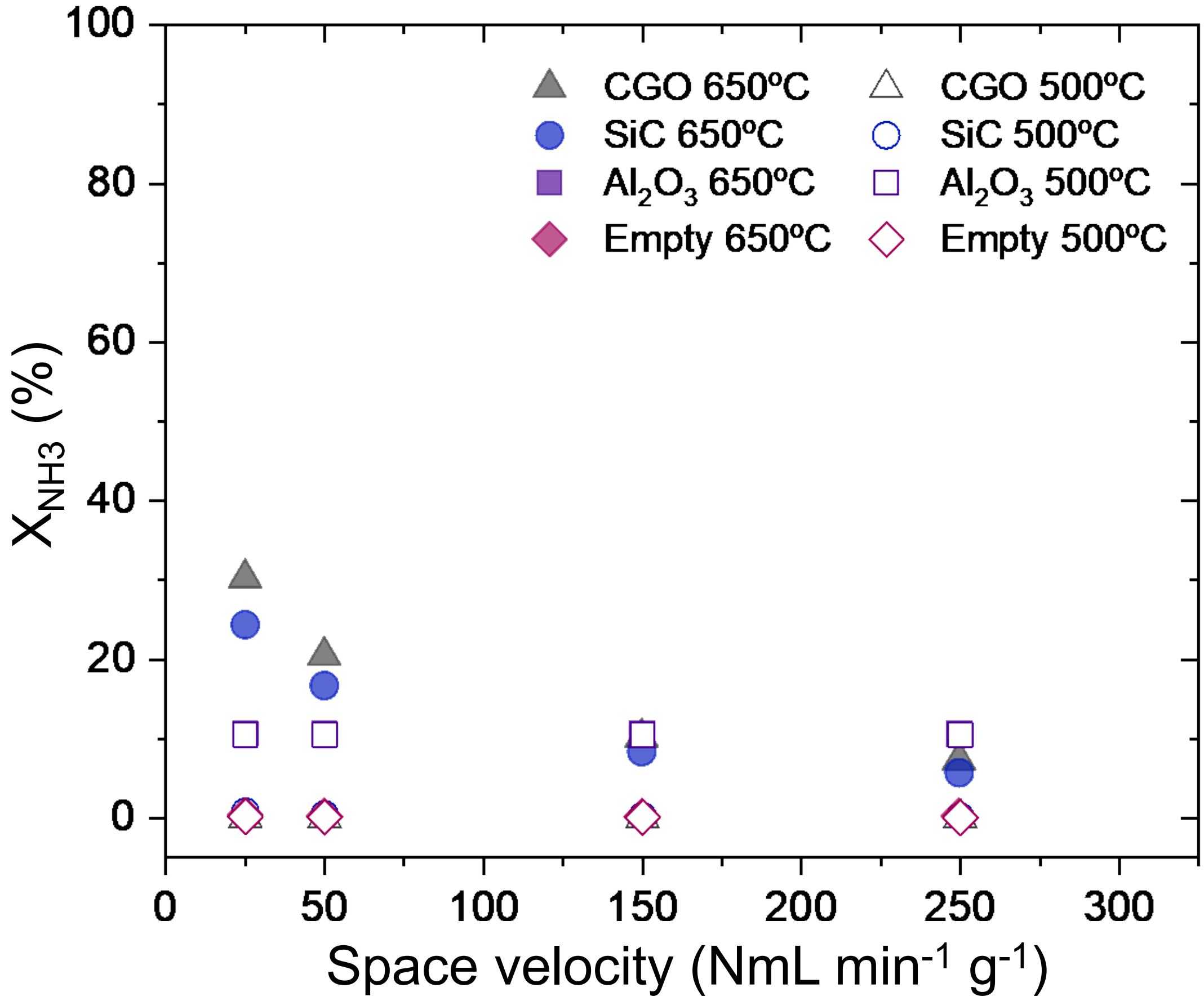

Figure S24. Thin reactor blank tests with silicon carbide, Al_2O_3 and empty compared to CGO at both 650 and 500 ºC. NH_3 conversion is represented as a function of flow rate and temperature variation.

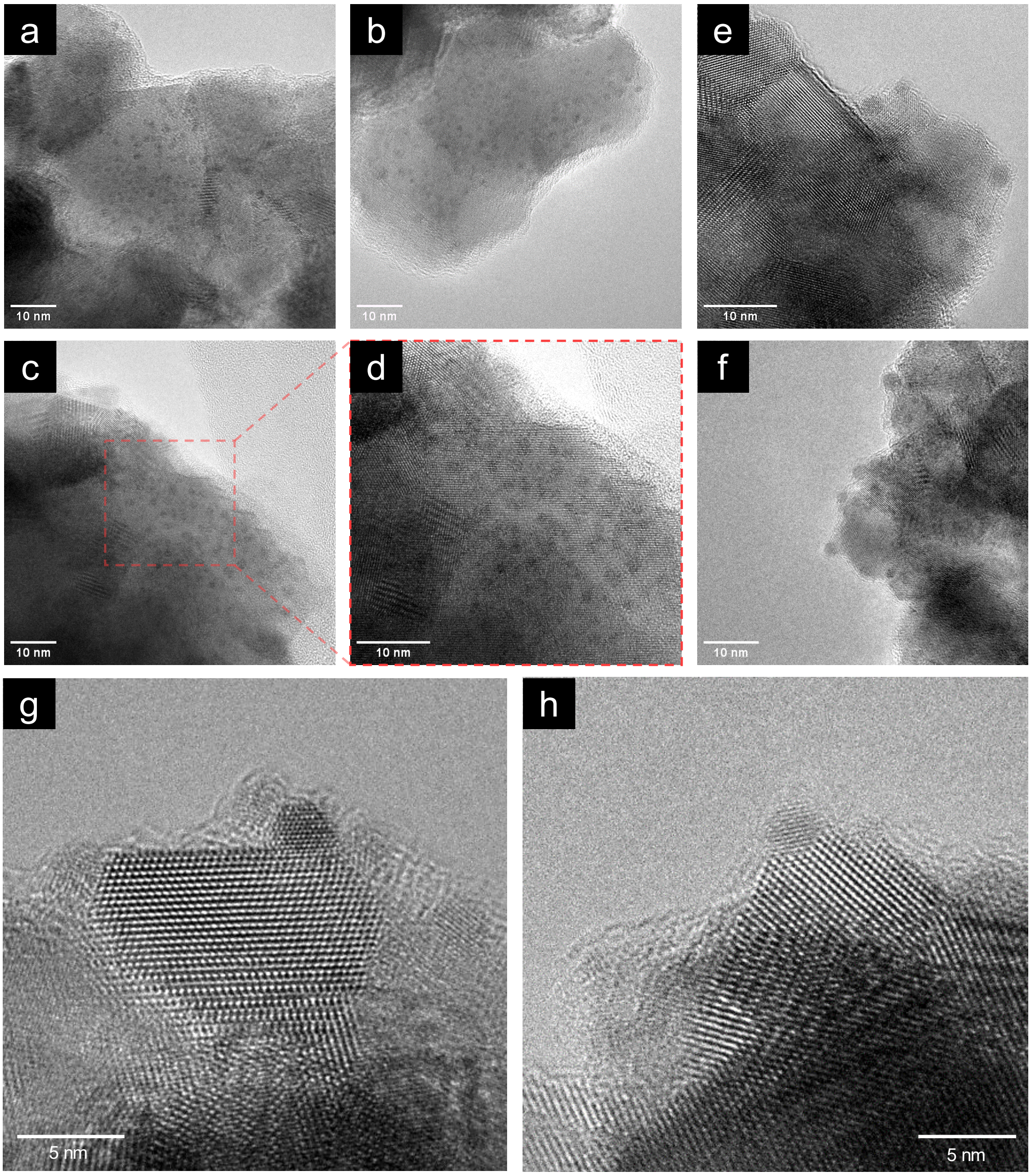

Figure S25. HRTEM micrographs of the exsolved CGORu after 260 h operation under NH_3 cracking conditions. For size determination (**Figure S26**), NPs were measured in bulk (**a-d**), crystal borders (**e-f**) and in higher detail (**g-h**).

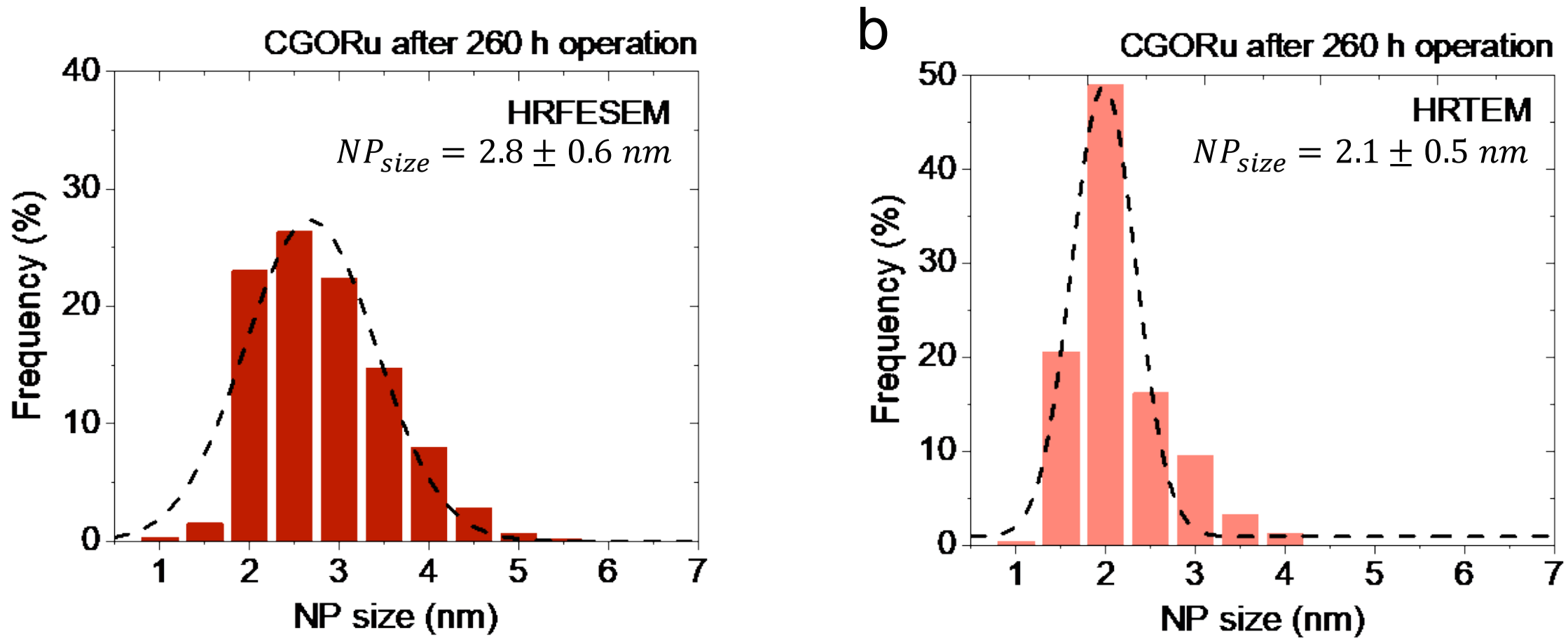

Figure S26. Size distribution histograms of the exsolved NPs from CGORu after 260 h operation under NH_3 cracking conditions, characterized through measured NPs by HRFESEM **(a)** and HRTEM **(b)**, showing no statistically significant differences between both methods and the exsolved material in terms of NP mean size.

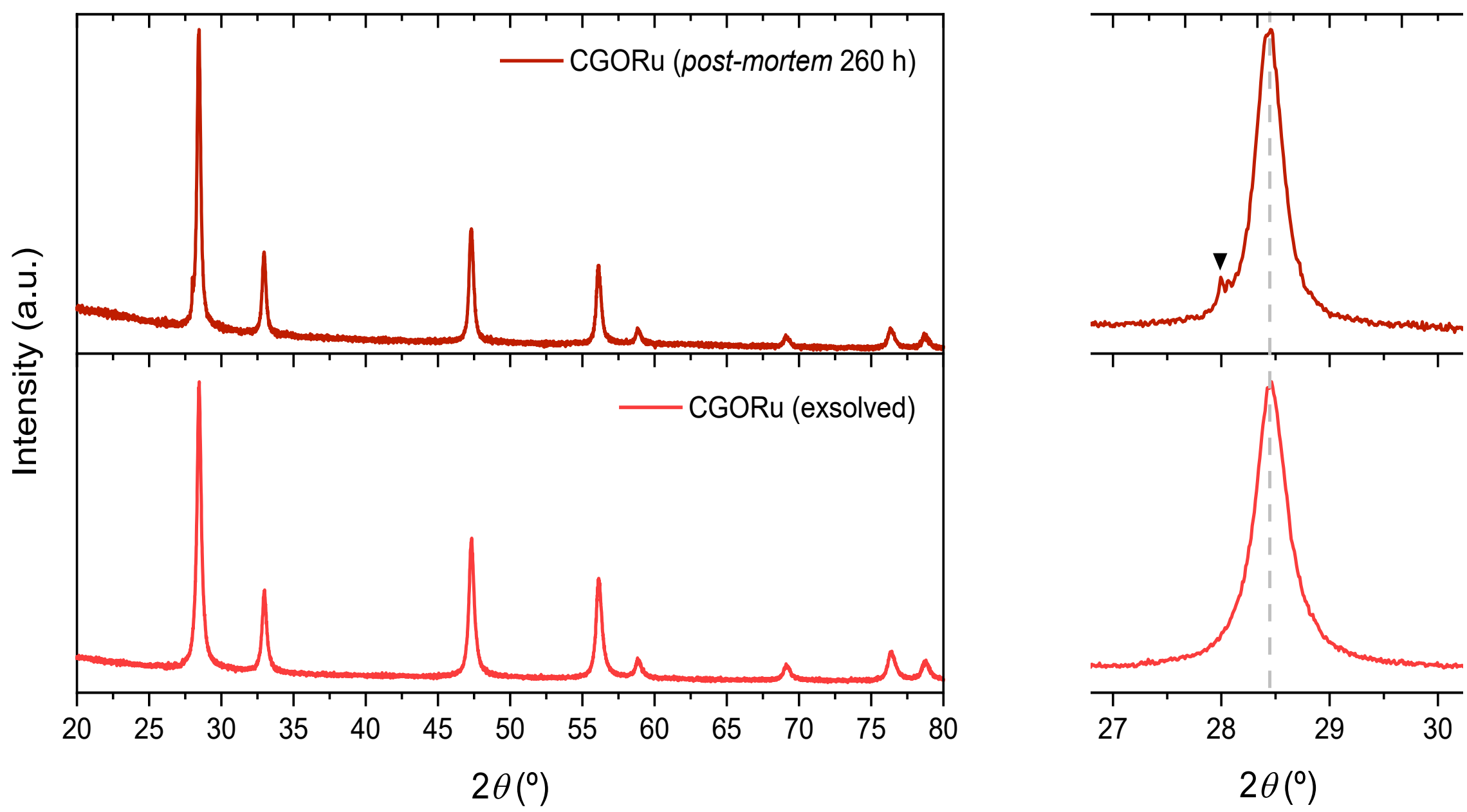

Figure S27. PXRD comparison between exsolved CGO before reaction tests and after 260-h NH_3 decomposition operation. No significant changes can be appreciated, highlighting the stability of the host oxide. The reflection at $2\theta \approx 28$ ° (black triangle) is attributed to the grease used in the zero-background sample holder.

References for Supporting Information

[1] M. Kim, G. Park, K. Jo, H. Lee, "Enhanced redox properties of Gd-doped CeO2–TiO2 induced by oxygen vacancies and disordered structure" *Mater. Today Chem.* **2023**, *29*, 101440.

[2] M. A. Paulin, G. Alejandro, D. G. Lamas, M. Quintero, R. O. Fuentes, J. E. Gayone, A. Butera, A. G. Leyva, J. Sacanell, "Oxygen vacancies and their role on the magnetic character of polycrystalline CeO2" *Ceram. Int.* **2023**, *49*, 5146–5153.

[3] H. He, C. Chen, C. Bian, J. Ren, J. Liu, W. Huang, "Enhanced Ammonia Decomposition by Tuning the Support Properties of Ni/GdxCe1-xO2-δ at 600 °C" *Molecules* **2023**, *28*, 2750.

[4] I. Lucentini, X. Garcia, X. Vendrell, J. Llorca, "Review of the Decomposition of Ammonia to Generate Hydrogen" *Ind. Eng. Chem. Res.* **2021**, *60*, 18560–18611.

[5] F. Koschany, D. Schlereth, O. Hinrichsen, "On the kinetics of the methanation of carbon dioxide on coprecipitated NiAl(O)" *Appl. Catal. B* **2016**, *181*, 504–516.

[6] S. Sayas, N. Morlanés, S. P. Katikaneni, A. Harale, B. Solami, J. Gascon, "High pressure ammonia decomposition on Ru–K/CaO catalysts" *Catal. Sci. Technol.* **2020**, *10*, 5027–5035.

[7] F. Hayashi, Y. Toda, Y. Kanie, M. Kitano, Y. Inoue, T. Yokoyama, M. Hara, H. Hosono, "Ammonia decomposition by ruthenium nanoparticles loaded on inorganic electride C12A7:e−" *Chem. Sci.* **2013**, *4*, 3124.

[8] J. Li, W. Wang, W. Chen, Q. Gong, J. Luo, R. Lin, H. Xin, H. Zhang, D. Wang, Q. Peng, W. Zhu, C. Chen, Y. Li, "Sub-nm ruthenium cluster as an efficient and robust catalyst for decomposition and synthesis of ammonia: Break the 'size shackles'" *Nano Res.* **2018**, *11*, 4774–4785.

[9] I. Lucentini, A. Casanovas, J. Llorca, "Catalytic ammonia decomposition for hydrogen production on Ni, Ru and Ni Ru supported on CeO2" *Int. J. Hydrogen Energy* **2019**, *44*, 12693–12707.

[10] W. Pyrz, R. Vijay, J. Binz, J. Lauterbach, D. J. Buttrey, "Characterization of K-Promoted Ru Catalysts for Ammonia Decomposition Discovered Using High-Throughput Experimentation" *Top. Catal.* **2008**, *50*, 180–191.

[11] K. McCullough, P.-H. Chiang, J. D. Jimenez, J. A. Lauterbach, "Material Discovery and High Throughput Exploration of Ru Based Catalysts for Low Temperature Ammonia Decomposition" *Materials* **2020**, *13*, 1869.

[12] Q. Su, L. L. Gu, A. H. Zhong, Y. Yao, W. J. Ji, W. P. Ding, C. T. Au, "Layered Double Hydroxide Derived Mg2Al-LDO Supported and K-Modified Ru Catalyst for Hydrogen Production via Ammonia Decomposition" *Catal. Letters* **2018**, *148*, 894–903.

[13] P. L. Bramwell, S. Lentink, P. Ngene, P. E. de Jongh, "Effect of Pore Confinement of $LiNH_2$ on Ammonia Decomposition Catalysis and the Storage of Hydrogen and Ammonia" *The Journal of Physical Chemistry C* **2016**, *120*, 27212–27220.

[14] A. Di Carlo, L. Vecchione, Z. Del Prete, "Ammonia decomposition over commercial Ru/Al2O3 catalyst: An experimental evaluation at different operative pressures and temperatures" *Int. J. Hydrogen Energy* **2014**, *39*, 808–814.

[15] D. Szmigiel, W. Raróg-Pilecka, E. Miśkiewicz, Z. Kaszkur, Z. Kowalczyk, "Ammonia decomposition over the ruthenium catalysts deposited on magnesium–aluminum spinel" *Appl. Catal. A Gen.* **2004**, *264*, 59–63.

[16] W. Raróg-Pilecka, "Ammonia decomposition over the carbon-based ruthenium catalyst promoted with barium or cesium" *J. Catal.* **2003**, *218*, 465–469.

[17] W. Raróg-Pilecka, D. Szmigiel, A. Komornicki, J. Zieliński, Z. Kowalczyk, "Catalytic properties of small ruthenium particles deposited on carbon" *Carbon N. Y.* **2003**, *41*, 589–591.

[18] J. Zheng, X. Liu, P. Xu, P. Liu, Y. Zhao, J. Yang, "Development of high pressure gaseous hydrogen storage technologies" *Int. J. Hydrogen Energy* **2012**, *37*, 1048–1057.

[19] D.-C. Huang, C.-H. Jiang, F.-J. Liu, Y.-C. Cheng, Y.-C. Chen, K.-L. Hsueh, "Preparation of Ru–Cs catalyst and its application on hydrogen production by ammonia decomposition" *Int. J. Hydrogen Energy* **2013**, *38*, 3233–3240.

[20] X. DUAN, J. ZHOU, G. QIAN, P. LI, X. ZHOU, D. CHEN, "Carbon Nanofiber-Supported Ru Catalysts for Hydrogen Evolution by Ammonia Decomposition" *Chinese Journal of Catalysis* **2010**, *31*, 979–986.

[21] F. R. García-García, J. Álvarez-Rodríguez, I. Rodríguez-Ramos, A. Guerrero-Ruiz, "The use of carbon nanotubes with and without nitrogen doping as support for ruthenium catalysts in the ammonia decomposition reaction" *Carbon N. Y.* **2010**, *48*, 267–276.

[22] S. J. Wang, S. F. Yin, L. Li, B. Q. Xu, C. F. Ng, C. T. Au, "Investigation on modification of Ru/CNTs catalyst for the generation of COx-free hydrogen from ammonia" *Appl. Catal. B* **2004**, *52*, 287–299.

[23] S. F. Yin, B. Q. Xu, S. J. Wang, C. F. Ng, C. T. Au, "Magnesia–Carbon Nanotubes (MgO–CNTs) Nanocomposite: Novel Support of Ru Catalyst for the Generation of COx-Free Hydrogen from Ammonia" *Catal. Letters* **2004**, *96*, 113–116.

[24] J. Guo, Z. Chen, A. Wu, F. Chang, P. Wang, D. Hu, G. Wu, Z. Xiong, P. Yu, P. Chen, "Electronic promoter or reacting species? The role of $LiNH_2$ on Ru in catalyzing NH_3 decomposition" *Chemical Communications* **2015**, *51*, 15161–15164.

[25] T. E. Bell, G. Zhan, K. Wu, H. C. Zeng, L. Torrente-Murciano, "Modification of Ammonia Decomposition Activity of Ruthenium Nanoparticles by N-Doping of CNT Supports" *Top. Catal.* **2017**, *60*, 1251–1259.

[26] P. Yu, J. Guo, L. Liu, P. Wang, F. Chang, H. Wang, X. Ju, P. Chen, "Effects of Alkaline Earth Metal Amides on Ru in Catalytic Ammonia Decomposition" *The Journal of Physical Chemistry C* **2016**, *120*, 2822–2828.
